# High-throughput sequencing analysis of nuclear-encoded mitochondrial genes reveals a genetic signature of human longevity

**DOI:** 10.1007/s11357-022-00634-z

**Published:** 2022-08-10

**Authors:** Brenda Gonzalez, Archana Tare, Seungjin Ryu, Simon C. Johnson, Gil Atzmon, Nir Barzilai, Matt Kaeberlein, Yousin Suh

**Affiliations:** 1 Department of Genetics, Albert Einstein College of Medicine, Bronx, NY 10461 USA; 2 Department of Pharmacology, College of Medicine, Hallym University, Chuncheon, Gangwon 24252 Republic of Korea; 3 Department of Medicine, Albert Einstein College of Medicine, Bronx, NY 10461 USA; 4 Department of Biology, Faculty of Natural Sciences, University of Haifa, Haifa, Israel; 5 Department of Pathology, University of Washington, Seattle, WA 98195 USA; 6 Department of Ophthalmology and Visual Sciences, Albert Einstein College of Medicine, Bronx, NY 10461 USA; 7 Departments of Obstetrics and Gynecology, and Genetics and Development, Columbia University, 630 West 168th Street, New York, NY 10032 USA

**Keywords:** Longevity, Mitochondria, Centenarian, Aging, Genetic variant

## Abstract

Mitochondrial dysfunction is a well-known contributor to aging and age-related diseases. The precise mechanisms through which mitochondria impact human lifespan, however, remain unclear. We hypothesize that humans with exceptional longevity harbor rare variants in nuclear-encoded mitochondrial genes (mitonuclear genes) that confer resistance against age-related mitochondrial dysfunction. Here we report an integrated functional genomics study to identify rare functional variants in ~ 660 mitonuclear candidate genes discovered by target capture sequencing analysis of 496 centenarians and 572 controls of Ashkenazi Jewish descent. We identify and prioritize longevity-associated variants, genes, and mitochondrial pathways that are enriched with rare variants. We provide functional gene variants such as those in *MTOR* (Y2396Lfs*29), *CPS1* (T1406N), and *MFN2* (G548*) as well as *LRPPRC* (S1378G) that is predicted to affect mitochondrial translation. Taken together, our results suggest a functional role for specific mitonuclear genes and pathways in human longevity.

**Supplementary Information:**

The online version contains supplementary material available at 10.1007/s11357-022-00634-z.

## Introduction

Geroscience research has been focused on understanding the molecular mechanisms driving the cellular and physiological functional decline that comes with increased age [1, 2]. Understanding the aging process to develop geroprotective interventions that extend health, quality of life, and independence during the final decades of life is one goal of geroscience research [2, 3]. Geroscience research has succeeded in identifying key biological pathways, labeled as “hallmarks or pillars of aging,” that drive the aging process [4, 5]. One of these hallmarks is the age-related dysfunction of mitochondria, found to contribute to biological aging across evolutionarily divergent species [6].

Mitochondrial dysfunction contributes to multiple physiological and functional deficits during aging, such as sarcopenia, frailty, reduced cardiac output, chronic inflammation (“inflammaging”), and cognitive decline, as well as numerous age-related diseases in humans and other animals [4, 5, 7]. Decreased mitochondrial function has been associated with overall age-related functional decline in nearly all tissues, especially those demanding higher energy, such as the heart, muscle, brain, and liver [8, 9]. Preservation of mitochondrial function through genetic or pharmacological measures has been shown to alter lifespan, the progression of aging, and age-related disease pathologies in model organisms [10–18]. For example, lifespan is extended in mice when mitochondrial uncoupling protein 2 (*UCP2*) [19] or mitochondrial catalase [20] is overexpressed. Somewhat paradoxically, lifespan can also be increased upon modest inhibition of mitochondrial electron transport chain (ETC) function, such as knockdown (KD) of the complex IV component *SURF1* in mice and in models of reduced ETC function in worms, yeast, and flies [21, 22]. The underlying mechanisms remain unresolved, and strong ETC inhibition uniformly causes severe disease and early death in both mice and humans. Mitochondrial function is also tightly interconnected with conserved longevity-assurance pathways that regulate aging in laboratory animals. These include nutrient sensing and metabolic regulation through the mammalian target of rapamycin (*MTOR*), insulin/insulin-like growth factor signaling (IIS), AMP-activated protein kinase (*AMPK*), and sirtuins [10, 23, 24].

Despite the extensive research in model organisms, it is still unclear whether the same genes and pathways in mitochondrial function are important for human longevity. The natural occurrence of genetic variants in humans that affect longevity offers an ideal starting point for addressing the knowledge gap. The best examples of such “natural mutants” are human centenarians, who managed to delay or ward off the diseases that normally begin to plague humans starting at middle age [25]. As demonstrated by multiple studies, this extreme human longevity has a strong genetic component and longevity is likely mediated via heritable resilience to age-associated diseases [26–31]. Given that the frequency of centenarians is only ~ 1/5000–7000 individuals [32], it has been hypothesized that genetic variation that contributes to exceptional longevity in centenarians is rare or absent in younger control populations [32, 33].

Identification of potentially protective rare variants in centenarians requires deep sequencing analysis of centenarian genomes [34–36]. Alternatively, targeted sequencing of candidate genes involved in the conserved pathways of aging provides an opportunity to test the relevance of candidate genes and pathways in human longevity. Indeed, by a comprehensive resequencing analysis of candidate genes focused on the well-established conserved pathways of aging, we have identified and characterized rare, functional variants in *IGF1R* [37, 38], *NFKBIA* [39], *CLU* [39], and *PRKCH* [39] that are associated with human longevity. While these variants are enriched in centenarians at a nominal significance (*p* < 0.05), functional analysis of these variants using cell models demonstrated parallel mechanisms between humans and model organisms. For example, the longevity-associated variants in the *IGF1R* gene cause reduced signaling through the insulin/insulin-like growth factor 1 signaling (IIS) pathway [37, 40], one of the major conserved pathways of aging [37, 38]. Furthermore, functional analyses of the longevity-associated gene variants in *NFKBIA*, *CLU*, and *PRKCH* suggest reduced signaling through the PKC/NF-κB pathways [39]. Thus, deep sequencing analysis of candidate genes in centenarians has the translational potential to reveal functional relevance of conserved genes and pathways on human longevity.

Here we describe an integrated functional genomics approach to identify rare functional variants in mitonuclear genes discovered by target capture sequencing analysis of centenarians and controls. We identify and prioritize candidate variants, genes, and mitochondrial pathways that are associated with human longevity and provide novel variants worthy of functional studies to investigate underlying mechanisms. Our results suggest that naturally occurring, potentially functional, rare DNA sequence variation in mitonuclear genes may contribute to human longevity and healthy aging by affecting mitochondrial function and homeostasis as has been demonstrated in model organisms.

## Methods

### Compiling and categorizing mitonuclear candidate genes for sequencing

The list of candidate genes to include for sequencing was compiled by comprehensive literature and database mining. The GeneCards Human Gene Database localization information [41] (https://www.genecards.org/), MitoCarta version 1 accessed 2014–2015 [42], and MitoMiner version 3 accessed 2014–2015 [43] (http://mitominer.mrc-mbu.cam.ac.uk/release-4.0/begin.do) were used to identify nuclear-encoded genes in the mitochondrial proteome. The Online Mendelian Inheritance in Man database was used to gather disease and phenotype information for candidate genes (OMIM®), accessed 2015 [44] (https://www.omim.org/). Human Ageing Genomic Resources (HAGR) [45] (https://genomics.senescence.info/), such as the Ageing Gene Database (GenAge, accessed 2015 and updated January 2019) and the Digital Aging Atlas (DAA) [46] (http://ageing-map.org/, accessed 2015 and updated January 2019), were used to identify and verify candidate genes associated with lifespan, aging, age-related disease, and other related traits in model organisms and humans.

Functional categorization of the candidate genes was done using a combination of detailed gene information from GeneCards gene summaries, Gene Ontology (GO) from the Reactome database [47] (https://www.reactome.org/), and Gene Ontology Resource [48] (http://geneontology.org/). Subcellular compartment localization scores were sourced from the Compartments database, which integrates numerous types of protein localization evidence from other sources and is linked through GeneCards (accessed January 2019, [49]) (https://compartments.jensenlab.org/Search).

### Sequencing and post-sequencing data annotation and summary statistics

Target capture next-generation pooled sequencing was conducted using equimolar concentrations of DNA samples from the well-established AJ longevity cohort of 494 cases (synonymous with probands or centenarians) and 572 controls [50]. The samples were combined as previously described [51] in 43 total pools for sequencing, each containing samples from 25 individuals per pool. The 43 pools were combined into 4 captured samples, with 11 pools per the first three captured samples and 10 pools in the fourth captured sample. Target capture probes were designed to enrich for all coding regions and some regulatory regions: exons, exon–intron junctions, 5′-untranslated and 3′-untranslated regions, and 2 kb upstream of the proximal promoter regions of candidate genes. The total target size for the mitonuclear candidate genes spanned ~ 4.6 Mb. The pool sequencing achieved a median coverage per pool of ~ 378.12-fold in total, which is equivalent to ~ 15-fold coverage per sample. Libraries were sequenced on the Illumina HiSeq2000 flow-cell at Axeq Technologies. The resulting data was processed at Einstein’s Genomics Computational Core. *P*-value was calculated by Fisher’s exact test. Final annotations including mapped gene, variant location, and variant impact were adjusted (unless otherwise stated) for each detected variant by Ensembl Variant Effect Predictor (VEP) (http://grch37.ensembl.org/info/docs/tools/vep/index.html) or by manually checking against the human reference genome (GRCh37/hg19) in the UCSC genome browser (University of California Santa Cruz Genomics Institute (https://genome.ucsc.edu/cgi-bin/hgGateway)) [52]. The variant ID annotations were updated with dbSNP144, accessed April 2016. The Manhattan plot was generated in R and all other summary statistics were calculated in Microsoft Excel. To determine the proportion of variants that are centenarian-enriched and common or centenarian-enriched and rare, the alternative (ALT) and reference (REF) alleles were first adjusted to ensure that ALT is the minor allele (MIN), followed by calculation of both MAF in each group and the cohort minor allele frequency (MAF), both based on observed diploid allele counts.

### GWAS analysis

The GWAS catalog from the National Human Genome Research Institute (NHGRI) and European Molecular Biology Laboratory-European Bioinformatics Institute (EMBL-EBI) (NHGRI-EBI) was mined for age-related trait associations with mitonuclear candidate genes (accessed 04/2016, http://www.ebi.ac.uk/gwas). Data was collected and compiled as described previously [53]. Briefly, all data for candidate mitonuclear genes reported in the GWAS catalog were extracted for analysis and categorized based on associated trait(s). Specifically, genes associated with age-related disease at *p* ≤ 10^−8^ and “lifespan,” “aging,” and “longevity” at *p* ≤ 10^−6^ were included for downstream analysis. All genes associated with diseases of related categories were grouped together (e.g., cardiovascular disease traits include “coronary heart disease,” “myocardial infarction,” “homocysteine levels,” and other disease terms and high confidence risk biomarkers that fit into the larger category of “cardiovascular disease”). If a gene and trait association from the same study (i.e., not a replication study) was reported multiple times, the association with the most significant *p*-value was retained for this analysis and duplicates were removed. From there, the number of associations and unique genes was quantified and graphed per category**.**

We used the recently developed R script for Enrichment of Age-Related Diseases in GWAS (EARDiG) https://github.com/biosinodx/EARDiG.

### Gene-based SKAT analysis

Gene-based rare variant analysis by Sequence Kernel Association Tests (SKAT) was accomplished through the R package “SKAT” [54]. SKATs were run twice, on sequencing data including and excluding singletons. The SKAT results reported are based on data excluding singletons as this is more stringent and there were few differences between SKAT results including and excluding singletons. Model organism lifespan data was extracted from GenAge, linked through HAGR using orthologs reported by GeneCards, the homologous human gene symbol, or Entrez IDs.

To ensure consistent results for GO enrichment on SKAT results, we compared against different reference backgrounds including the whole human genome (~ 21,000 genes), the mitochondrial proteome (~ 2220 genes), and the mitonuclear candidate list (~ 660 genes). We used the Protein Analysis Through Evolutionary Relationships database (PANTHER, http://pantherdb.org/).

We defined a comprehensive annotation criteria to filter top variants of interest for in silico prediction: (1) single-variant association signal with longevity (*p* ≤ 0.05); (2) directionality of enrichment, with higher priority for those that are enriched in centenarians compared to controls (i.e., observed MAF_cases_ > observed MAF_controls_); (3) location, with higher priority for nonsynonymous variants (nsVs) over non-coding variants (ncVs); (4) impact, with higher priority for nsVs with non-conserved (NC) biochemical and physiochemical amino acid changes, and damaging or deleterious predictions, and higher priority for ncVs with strong Regulome database scores, that fall within predicted regulatory regions as assessed by manual checking of DNase hypersensitivity, promoter-associated and enhancer-associated histone marks, and TF-binding evidence in the UCSC genome browser ensemble track hubs [55]; (5) validation by genotyping and other population MAF information obtainable for further confidence—global MAF from 1000Genomes, average MAF from GnomAD (~ 15,000 whole genomes and ~ 125,000 exomes) and Exac (~ 60,000 exomes) and MAF from the Geisinger and Regeneron whole-exome sequencing study of the DiscovEHR cohort containing ~ 50,700 individuals [56]; and (6) comprehensive annotations showing a priori biological evidence of phenotypes associated with the SNP- or its respective gene in humans or model organisms including evolutionary conservation at the variant site, protein domains or post-translational modifications mapped at or near the variant site, gene-associated or SNP-associated phenotype information including gene expression changes with age in humans or model organisms and model organism longevity information.

Many sources were used in conjunction with the Genome Reference Consortium Human Build 37 to obtain and corroborate annotations for variant filtering. Ensembl VEP [57] was used as a source of variant position information, global or average control MAF information from the 1000Genomes Project, gnomAD, and ExAC and prediction scores for protein impact of nsVs, including Condel. The Model organism Aggregated Resources for Rare Variant ExpLoration (MARRVEL) was a source of variant site evolutionary conservation in model organisms, CADD protein impact prediction scores, gnomAD and ExAC population allele frequencies, and SNP-associated human phenotype ontology (HPO) data from the sequencing of ~ 10,500 exomes at the University of Washington’s Mendelian Genetics Center via the linked source—Geno_2_MP (Marrvel.org) [58].

The UCSC genome browser was used with track hubs to annotate gene and SNP associations (Genetic Association Database of Complex Disease and Disorders (GAD), The NHGRI-GWAS Catalog, clinically associated SNPs by Flagged SNPs in dbSNP144, and the Genomenon Mastermind track for literature mining of genetic variants). ENCODE tracks hubs on UCSC genome browser were used to annotate regulatory regions (ENCODE/broad chromatin state segmentation, DNase hypersensitivity cluster, histone modifications, and transcription factor ChIP-seq, and the Genetic Information Research Institute RepeatMasker track for overlapping elements such as LINEs/SINEs) [59]. The UCSC genome browser was also used with tracks to annotate protein information such as domains or structures, modifications, sequences, and alignments (UniProt Protein annotations, and PeptideAtlas MassSpec peptide sequence).

Amino acid biochemical conservation was manually annotated as conservative (‘C), non-conservative (NC), or radical (‘R) based on information about amino acid R-group properties including charge, polarity, hydropathy, and size [60–64] (https://en.wikipedia.org/wiki/Proteinogenic_amino_acid). Amino acid substitutions designated “C” have zero biochemical property differences, while “NC” substitutions have at least 1 biochemical difference, and “R” substitutions have ≥ 2 changes including a change in size which is regarded as more radical change because of the potential for steric hindrance.

Prediction annotations of the likelihood that a polymorphism is neutral or damaging to protein function were determined using two independent, experimentally validated algorithms that automatically provide prediction scores based on multiple lines of evidence for such polymorphisms: the CONsensus DELeteriousness score of missense mutations (Condel) [65] and the Combined Annotation Dependent Depletion scores (CADD, CADDphred) [66, 67]. When the reference and alternative amino acids are “C,” the biochemical and physiochemical characteristics are more neutral and reduce the likelihood of a functional protein change; e.g., the reference and alternative amino acids are of similar size, charge, or polarity so the biochemical change is not severe and the expectation is that Condel and CADD would predict the SNP to be “neutral or benign,” whereas “NC” or “R” substitutions infer that the properties of the two amino acids differ enough to potentially alter protein structure and/or function and the Condel and CADD predictions would be “probably or possible damaging or deleterious.”

### Genotyping

High-throughput iPLEX mass array genotyping of 112 variants was conducted at the Einstein Genomics Core using amplified DNA for 1083 AJ cohort individuals. Genotyping analysis and variants were validated based on comparison of allele frequencies between pooled sequencing and genotyping. A total of 100 of the variants were successfully genotyped, while 12 variants failed at primer design. Thirty-four variants failed to validate for technical and quality reasons.

### Protein modeling

The 2D protein domain mapping and schematic was created using data and figures modified from feature viewers on UniProt [68] (https://www.uniprot.org/) and PDB [69] (The Protein Data Bank, rcsb.org) for the LRPPRC protein (ID: P42704). The 3D rendering of the partial structure of LRPPRC is a homology-based model generated from a template (5nnr.1.A) based on a protein with similar sequence (i.e., homology model) using the “Build Model” function with the FASTA sequence for the full 130 kDa protein (UniProtKB/Swiss-Prot: P42704.3) on SWISS-MODEL ExPASy [70] (https://swissmodel.expasy.org/). This was the best choice homology model that encompassed the C-terminal region of interest in our protein as no crystal structure for LRPPRC has been resolved yet. The University of California San Francisco’s (UCSF) Resource for Biocomputing, Visualization, and Informatics (RBVI) Chimera tool was used to depict and manipulate the tertiary structure of the homology model, generate graphics, and analyze and calculate distances between nearest neighboring atoms and residues and between potential hydrogen bonds [71].

## Results

### Selection of candidate aging-related mitonuclear genes

**Fig. 1:**
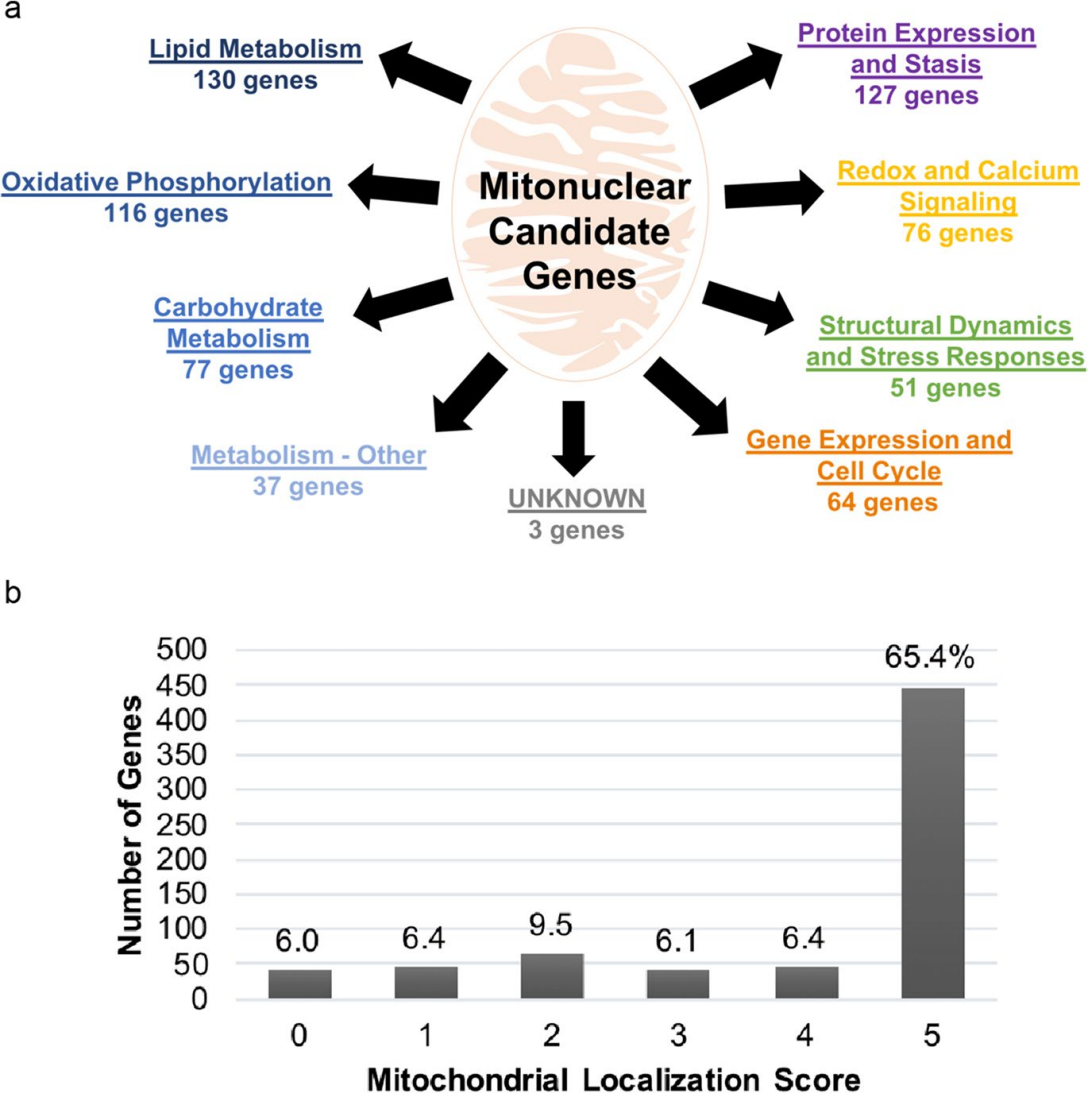
Mitochondrial functions and localization score of selected mitonuclear candidate genes. **a** Nuclear-encoded genes with mitochondrial functions placed in respective functional categories. Candidates chosen based on evidence for association with aging or longevity in model organisms or humans. **b** Localization scores were retrieved from Compartments, linked through GeneCards. Increasing localization score indicates increasing confidence that the gene is localized to the subcellular compartment (0—lowest confidence, 5—highest confidence score for mitochondrial localization)

Candidate nuclear-encoded mitochondrial genes (mitonuclear genes) were chosen on the basis of biological evidence for their function in mitochondria, aging processes, age-related diseases, and longevity or lifespan in model organisms or humans (see the “Methods” section). We excluded genes encoded by the mitochondrial genome (mtDNA) from our candidate list in order to focus specifically on mitonuclear gene variants. In total, ~ 680 mitonuclear genes were chosen as candidates for the target capture sequencing-based discovery of longevity-associated variants (LAVs) and genes (LAGs). There are many mitochondrial pathways that are important in aging and age-related pathology; thus, we were interested in how our large list of candidates represented these mitochondrial functions, which could be useful in downstream analysis. We divided all mitonuclear candidates into categories according to their respective functions and pathways within and around the mitochondria, according to GO and detailed gene summaries provided by GeneCards (Fig. 1a and Tables S1, S2). As expected, many of the candidate genes have primary functions in metabolism; however, the mitonuclear candidate genes also represent a diversity of mitochondrial functions in cellular homeostasis. The categories representing the most candidate genes, with > 100 genes per category, are lipid metabolism, oxidative phosphorylation, protein expression, and proteostasis. Although not a requisite, 78% of our candidate genes have high mitochondrial localization scores (≥ 3; with 65% having the highest confidence score of 5) as indicated in GeneCards and Compartments (Fig. 1b).

### Characterization of candidate mitonuclear genes by GWAS-detected phenotypes

Common human genetic variation associated with different phenotypes and traits, including disease risk variants and lifespan-associated variants, can be sourced from published GWAS data. Lifespan-associated variants are defined by trait designations including “aging (time to death/event),” “lifespan,” “longevity (85 +),” and “parental longevity.” We utilized GWAS data to further annotate biological information, in the context of human aging and age-related disease, to our mitonuclear candidate genes. To identify the number of age-related disease and lifespan associations with our candidate genes, we extracted the data pertaining to mitonuclear candidate genes and age-related diseases from the GWAS Catalog using a significance threshold of *p* < 5 × 10^−8^ for disease associations, and *p* < 5 × 10^−6^ for lifespan associations. In total, ~ 16% of the 659 mitonuclear candidates harbor common variants associated with human age-related disease (Fig. S1a) or lifespan in GWAS (Fig. S1b). Metabolic disease contains the greatest number of associations, while inflammatory/immunological diseases are associated with the greatest number of unique mitonuclear genes. The most common candidate genes associating with multiple disease categories were *LIPC* and *FADS1/2*, which are lipid metabolism genes, and *CPS1*, which is important for amino acid metabolism and the urea cycle. Only 6 candidates were associated with lifespan terms: *SUCLA2*, *ECHS1*, *PARKIN*, *CYP51A1*, *PFKM*, and *TOMM40*-*APOE*. Using our newly developed tool (EARDiG) for determining if genes within a query set are statistically enriched in GWAS of age-related diseases (“Methods” section), it was determined that the mitonuclear candidates are significantly enriched in metabolic diseases (*p* = 0.018) (Fig. S1c) but not in any other category (Fig. S1d).

### Discovery of genetic variants in candidate mitonuclear genes

To efficiently sequence all possible variants, we previously established a method of next-generation sequencing on pooled samples with probes designed to capture specific DNA targets [51, 72]. This method of sequencing permits the capture of rare coding and regulatory variants. Samples sequenced are from 496 probands and 572 controls of the AJ Longevity Genes Project [73]. Proband or case samples include centenarians with an average lifespan ≥ 95 years, while control samples come from individuals with no family history of longevity whose parents lived to a maximum of 85 years and are often spouses of probands’ offspring allowing further control for the environment and lifestyle.

**Fig. 2:**
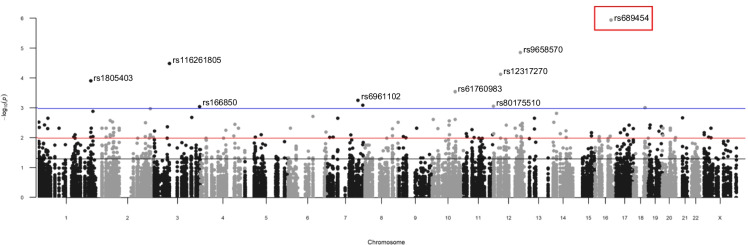
Significant gene variants discovered by pooled sequencing. Manhattan plot representing all 66,419 variants discovered as individual dots, with association signal significance (-log10 (*p*-value)) with longevity in the AJ cohort as a function of chromosomal location. Blue line is *p* = 0.001, red line is *p* = 0.01, and black line is *p* = 0.05. Red box highlights the most significant NQO1 variant. The majority of variants are novel (~ 78% as determined by Ensembl VEP and based on lack of variant IDs from dbSNP144)

In total, 66,419 variants were sequenced and annotated to ~ 659 of our 683 mitonuclear candidate genes. Twenty-four genes were removed after quality control or because no variation was detected in those target regions, suggesting high sequence conservation. Due to the lack of statistical power that often restricts single-variant analysis in rare variant association studies, no variants reached multiple testing correction significance except the most significant common variant, rs689454, located upstream of the *NQO1* gene with an association of *p* = 1 × 10^−6^ (FDR = 0.050) (Fig. 2). The top 10 variants are located in the following genes (in order of highest to lowest significance): *NQO1*, *NOS1*, *SUCLG2*, *DNM1L*, *PARP1*, *PI4K2A*, *EIF4G2*, *NRF1*, *ZNF746*, and *FOXM1*.

**Fig. 3:**
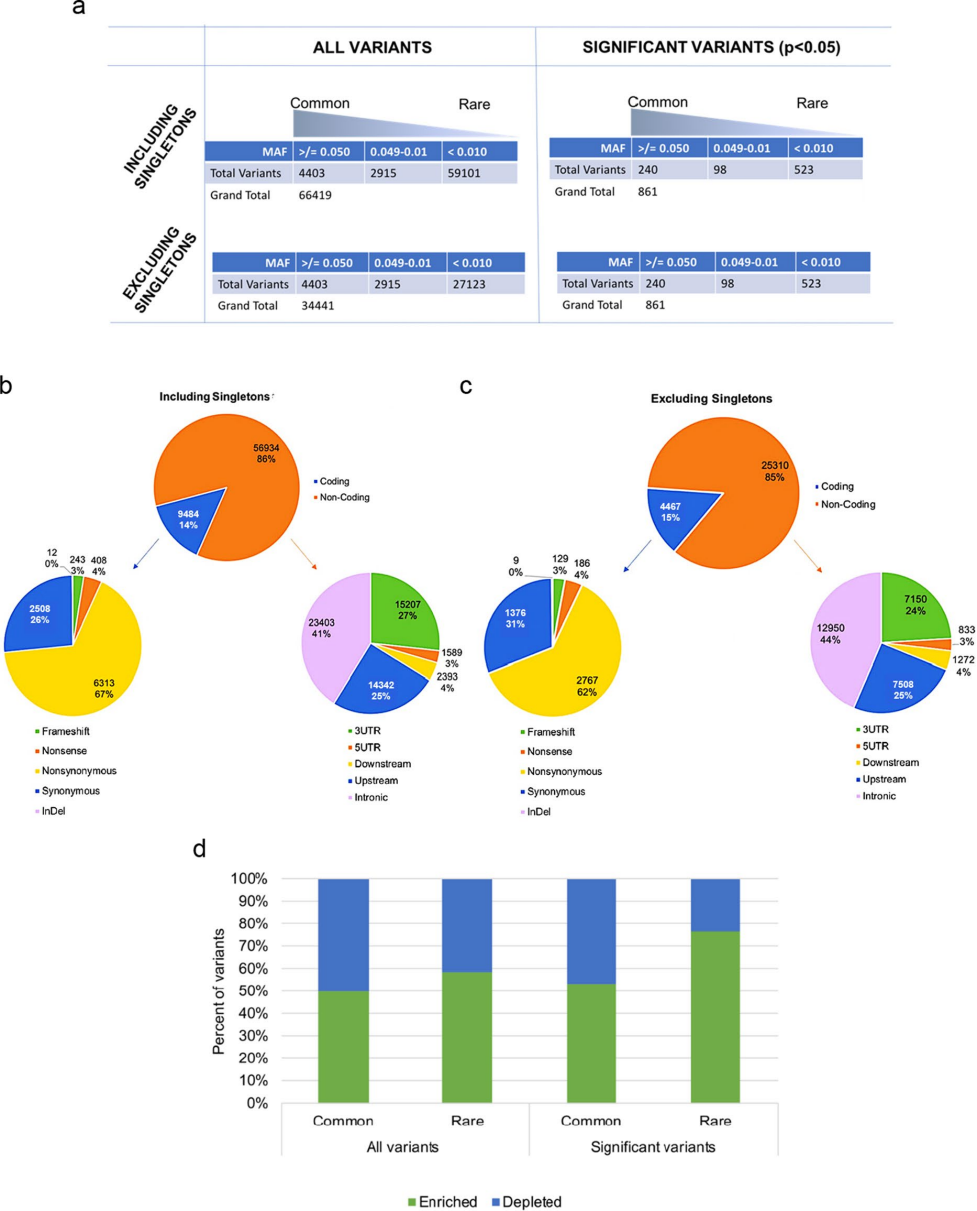
The distribution and characteristics of mitonuclear gene variants discovered by pooled sequencing. **a** The relationship between singleton variants, allele frequency, and association significance in sequencing data. Singleton variants account for approximately half of the variants identified by sequencing. **b** Distribution and percentages of different types of genetic variations as annotated by genic location (exonic/coding, non-exonic/non-coding) and impact. Pie charts express both numbers and the percent of variants in the respective pie. **c** Distribution of different types of genetic variations excluding singletons as annotated by genic location (exonic/coding, non-exonic/non-coding) and sequence impact (i.e., 1% less ncVs, 1% more cVs: 3% less 3′UTR, 3% more intronic, and 5% less nsVs). Pie charts express both numbers and the percent of variants in the respective pie. **d** The proportion of centenarian-enriched to centenarian-depleted variants is based on MAF in cases versus MAF in controls and separated based on observed cohort MAF. Common = MAF > 0.010. Rare = MAF ≤ 0.010. Green bars = percent of variants enriched in centenarians, blue bars = percent of variants depleted in centenarians. The analysis was done after excluding singletons

The majority of detected variants are novel (~ 78% as determined by Ensembl VEP and based on lack of variant IDs from dbSNP144); however, nearly half of the total variants are singletons, representing a large portion of the novel variant pool that can be detected by deep sequencing (Fig. 3a). The total number of variants with a nominal association *p*-value ≤ 0.050 is 854, of which ~ 40% (328) are common (MAF > 0.010) and ~ 60% (526) are rare (MAF ≤ 0.010). As expected, the proportion of non-coding (ncVs) or non-exonic variants exceeds that of coding (cVs) or exonic variants. Among cVs, 67% are nonsynonymous (nsVs), 26% are synonymous (sVs), 4% are stop gain (sg or nonsense), 3% are frameshifts (fs), and < 1% are in-frame insertions or deletions (InDels.) (Fig. 3b). Excluding singletons does not substantially change the proportions of the different types of variants (Fig. 3c).

When a variant has a greater MAF in centenarians, as compared to controls, we call the variant “enriched” with regard to centenarians and “depleted” in controls. When considering all variants (regardless of *p*-value), the proportion of variants enriched and depleted in centenarians remains ~ 50% (Fig. 3d). However, when only significant variants (*p* ≤ 0.050) are considered, the proportion of rare variants that are enriched in centenarians is far greater. Nearly 80% of rare variants are enriched in centenarians and the remaining 20% are depleted (Fig. 3d). In addition, we wanted to know if centenarians harbor more of a specific type of variant, e.g., do they have more cVs or ncVs compared to controls? The distributions of different types of variants between centenarians and controls do not differ very much (Fig. S2a). Excluding singletons only alters the proportions of some types of variants by no more than 5% (e.g., there are 5% less nsVs in total, and centenarians have 1% more frameshifts compared to controls after excluding singletons) (Fig. S2b and S3a, b).

### Identifying longevity-associated mitonuclear genes and pathways by gene-based analysis

**Fig. 4:**
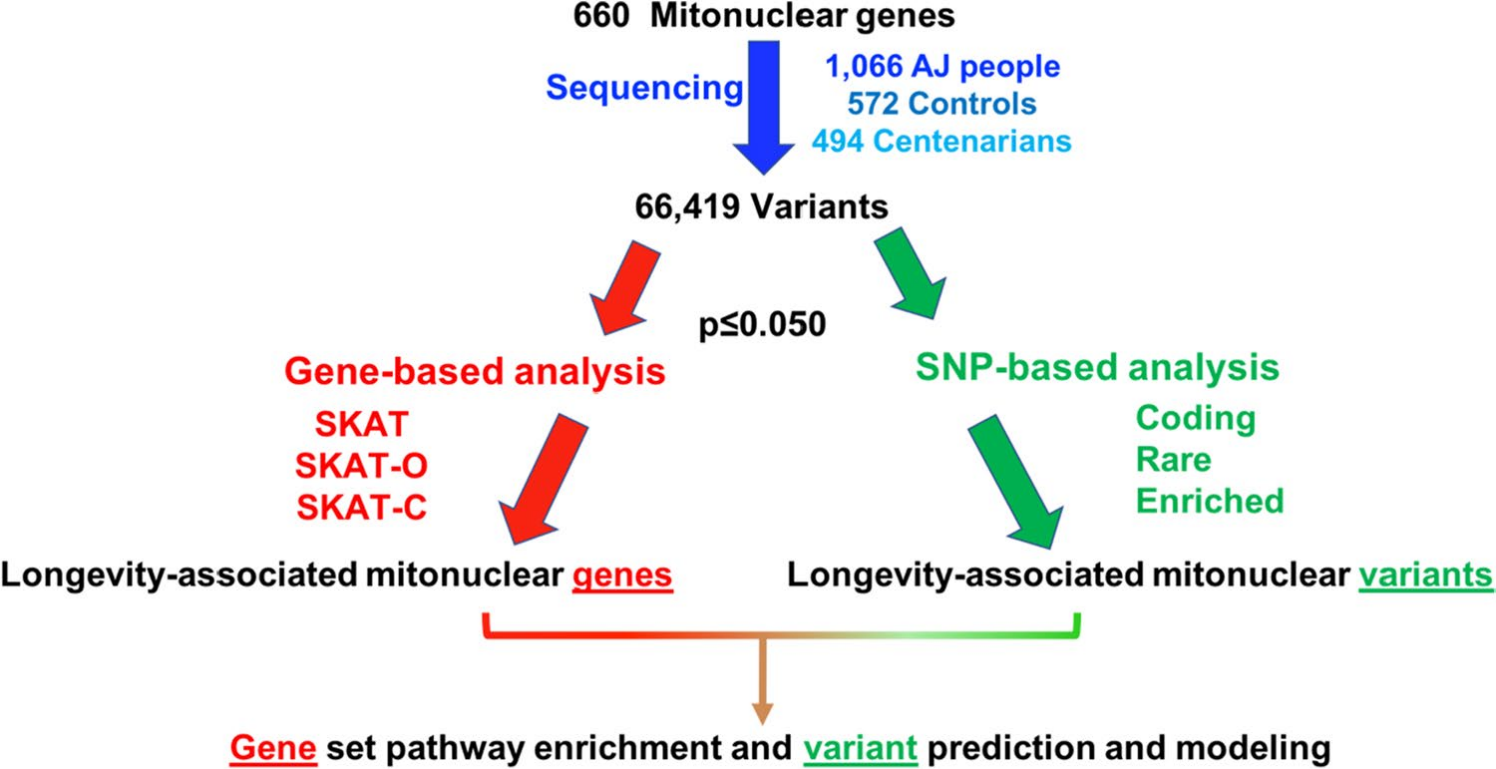
Workflow to filter, identify, and analyze longevity-associated mitonuclear genes and variants. Gene-based variant aggregate analyses by SKATs were used to identify longevity-associated genes, while individual SNP-based analysis was performed by screening variants meeting certain criteria of interest: mainly nsVs, rare variants (MAF ≤ 0.010), and centenarian-enriched variants were filtered for subsequent downstream analyses, including gene set pathway enrichment, variant effect prediction, and modeling in silico

We applied a workflow to variants discovered by target capture sequencing to systematically filter top longevity-associated genes (LAGs), variants (LAVs), and pathways (LAPs). It has been done under the hypothesis that rare centenarian-enriched variants may be protective and functionally causal in longevity compared to common variants and that these causal rare variants may or may not function in aggregate (Fig. 4). Gene-based analyses can help overcome rare variant statistical challenges by treating all variants within a single locus as a group to measure their aggregate longevity-association statistic. Thus, data were filtered to identify LAGs by using three types of Sequence Kernel Association Test (SKAT, SKAT-C, and SKAT-O), a rare variant burden test for joint significance of multiple genetic variants within a single gene [54]. Identifying genes that are significant across all SKATs or in specific SKATs can be informative for downstream interpretation because SKATs analyze different information: SKAT is specific for rare/low-frequency variants, SKAT-C includes common variants as well as rare variants, and SKAT-O gives more weight to variants that are strongly enriched in one group over another. Hence, with SKAT-O, we can assess the significance of overall directionality among cases and controls for all variants within a locus (i.e., whether variants in a given gene are significantly enriched in AJ centenarians).

Gene-based analysis revealed 76 nominally significant (*p* ≤ 0.050) longevity-associated genes, and we call these 76 genes the “longevity-associated gene set” (Tables S3 and S4). Mitochondrial genes well-known for their associations with aging, age-related disease, and lifespan, including *PPARG*, *TP53*, *PARP2*, and *APIP*, were found to be significantly associated with AJ longevity. However, none of the genes is significant after multiple test correction (Bonferroni, 660 tests, *p* = 7.5 × 10^−5^). Nonetheless, by incorporating biological evidence, we can gather further support for the genes that are more likely to be true positives. Thus, we further analyzed and annotated the longevity-associated gene set in terms of their representative mitochondrial functions and pathways, and their aging biology context, by data and text mining.

**Fig. 5:**
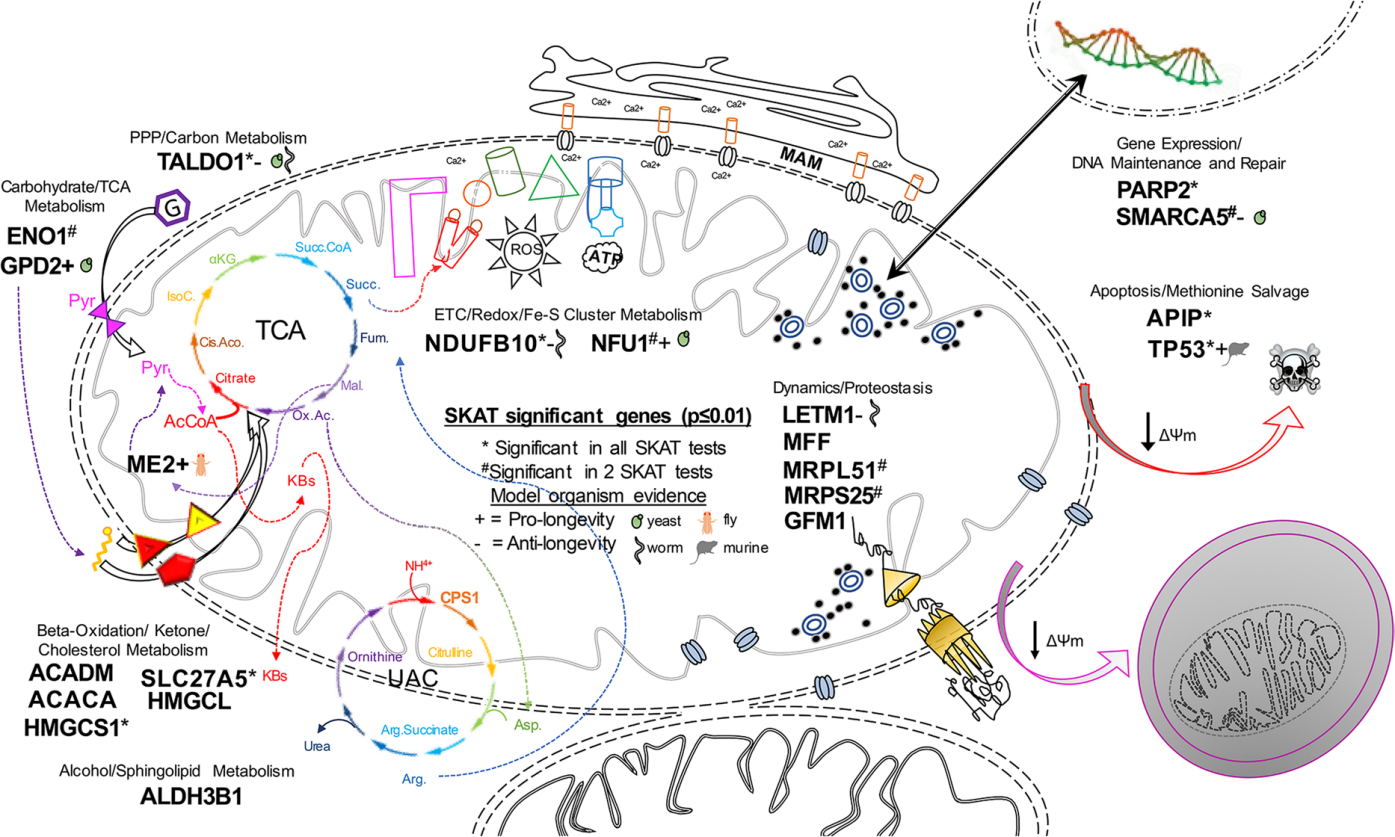
Longevity-associated genes and pathways discovered by gene-based association study. Mitonuclear genes with the greatest significance (*p* ≤ 0.01) in at least one SKAT are shown in their respective functional pathway(s); genes are not significant after MTC. A pro-longevity gene indicates evidence that overexpression of the gene can increase lifespan or a loss of function can decrease lifespan in the indicated model organism; an anti-longevity gene indicates evidence for overexpression decreasing lifespan or loss of function increasing lifespan in the indicated model organism. ΔΨm = mitochondrial membrane potential; Pyr = pyruvate; AcCoA = acetyl-coA; PPP = pentose phosphate pathway; TCA = the citric acid cycle; Cis.Aco. = cis-aconitase; IsoC. = isocitrate; α-KG = alpha-ketoglutarate; Succ.CoA = succinyl-CoA; Succ. = succinate; Fum. = fumarate; Mal. = malate; Ox.Ac = oxaloacetate; ETC = electron transport chain; Fe-S = iron-sulfur; MAM = mitochondrial ER-associated membrane; ROS = reactive oxygen species; ATP = adenosine triphosphate; KBs = ketone bodies; UAC = uric acid cycle; NH4 +  = ammonia; Arg. = arginine; Asp. = aspartate; Arg.Succinate = argininosuccinate; CPS1 = carbamoyl phosphate synthase

A summary schematic of the 21 top (*p* ≤ 0.010) longevity-associated mitonuclear genes illustrates the mitochondrial pathways represented, the experimental evidence for regulation of lifespan in model organisms, and the overlapping genes found to be significant in multiple SKATs (Fig. 5). For example, *LETM1*, a mitochondrial inner membrane calcium and proton (Ca^2+^ /﻿H^+^) antiporter that also regulates mitochondrial morphology and dynamics, and which is SKAT-O significant (*p* = 0.0026) in our analysis, is reported to be an anti-longevity gene in *Caenorhabditis elegans* due to the 21% extension of mean lifespan that is observed after *letm-1* RNAi-mediated knockdown [74]. We identified 46 variants annotated to *LETM1*: 37 as rare, 25 enriched in centenarians, 23 both enriched and rare, and 35 ncVs, with the majority (32) mapping to the 3′UTR of *LETM1*.

Using the PANTHER tool for GO analysis, we assessed the enrichment of mitochondrial pathways in the longevity-associated gene set for different reference backgrounds (Table S5). The fold enrichment of ketone body metabolism in the longevity-associated gene set was significant—passing multiple test correction against all backgrounds except the most stringent mitonuclear candidate list, as expected. There are ~ 12 ketone metabolism genes reported by PANTHER in the human reference list, and half were included in the candidate gene list (*AACS*, *ACAT1*, *BDH1*, *HMGCL*, *HMGCS2*, and *SLC27A5*), representing < 1% of the total mitonuclear candidate genes and all except 1 (*ACAT1*) are longevity-associated by SKATs (*p* ≤ 0.050). *SLC27A5*/*FATP5*, a liver-specific transporter of bile and fatty acids for lipid uptake and bile-acid synthesis, and *HMGCL*, the lyase catalyzing the conversion of HMGCoA to the ketone body acetoacetate, are among the top significant longevity-associated ketone metabolism genes, with *SLC27A5* showing significance in all SKATs (*p* ≤ 0.035). However, 2 other top significant genes (*ACACA* and *HMGCS1*) are not annotated to ketone metabolism in PANTHER but do play roles in the pathway, suggesting that the enrichment of ketone metabolism is actually under-represented by PANTHER GO analysis. These genes may not be annotated to “ketone metabolism” because *ACACA* and *HMGCS1* have primary roles in fatty-acid beta-oxidation which is an important upstream step to producing AcCoA substrates for hepatic mitochondrial ketone synthesis, and mevalonate substrates for cholesterol synthesis, which can occur as a non-oxidative fate of ketone bodies, respectively [75, 76].

### Prioritizing potentially functional longevity-associated mitonuclear variants

To identify functional longevity-associated variants, we further prioritized rare LAVs (Fig. 4) on the basis of their functionality of coding variants predicted by CADD and Condel algorithms for variant deleteriousness impact scores and enrichment in centenarians as compared to controls (see the “Methods” section)***.*** As a result, we identified 112 longevity-associated variants in 86 genes with different annotations (Table S6) and identified 59 nsVs (Table S7). The majority of prioritized variants are centenarian-enriched (~ 88%; 90) and rare (63%), 31 and 26 of which fall into regions of high vertebrate conservation and in conserved residues in invertebrates (yeast, worms, or flies), respectively. Among the 59 prioritized nsVs (Table S7), 54 resulted in not conserved amino acid substitutions, 14 in Radial (R) substitutions, and Condel predicted 21 to be deleterious, while 29 are reported possibly—likely damaging by CADD and are therefore very strong candidates. Given the rarity of LAVs (Table S7), we performed genotyping analysis to validate 65 LAVs without any technical and quality issues, of which 30 nsVs were predicted to be functional—18 LAVs by CADD and 12 LAVs by Condel, representing our strongest candidate variants (Table S8).

We also prioritized potentially functional longevity-associated genes (LAGs). Genes containing multiple top nsVs could represent “hotspots” for rare, potentially functional longevity associations. Thus, we note that 5 genes harbor multiple prioritized nsVs: *ACACB*, *ALDOA*, *ATP5S*, *MRPL51*, and *LRPPRC*. These genes are involved in various mitochondrial functions: *ACACB* encodes the key lipid synthesis enzyme that carboxylates acetyl-CoA to malonyl-CoA; *ALDOA* is the muscle-type isoform of the glycolytic aldolase enzyme that catalyzes the fourth step of glycolysis—the condensation of 6-carbon fructose 1,6-bisphosphate to 3-carbon glyceraldehyde 3-phosphate; *ATP5S/DMAC2L* encodes a subunit of the membrane and proton channel component (Fo) in the terminal ETC complex mitochondrial ATP synthase (complex V); *MRPL51* encodes a protein component of the large 39S mitochondrial ribosomal subunit; and *LRPPRC/LRP130* (leucine-rich pentatricopeptide repeat containing 130 kDa protein) encodes an RNA-binding protein that primarily positively regulates mitochondrial mRNA stability and translation. Furthermore, LAVs in the *CPS1* (rs1047891; T1406N), *MFN2* (G548*), and *MTOR* (Y2396Lfs*29) have been implicated in longevity, age-related disease, and endophenotypes associated with lifespan determination (see the “Discussion” section).

### Modeling and predicting biological impact of LRPPRC variants

**Fig. 6:**
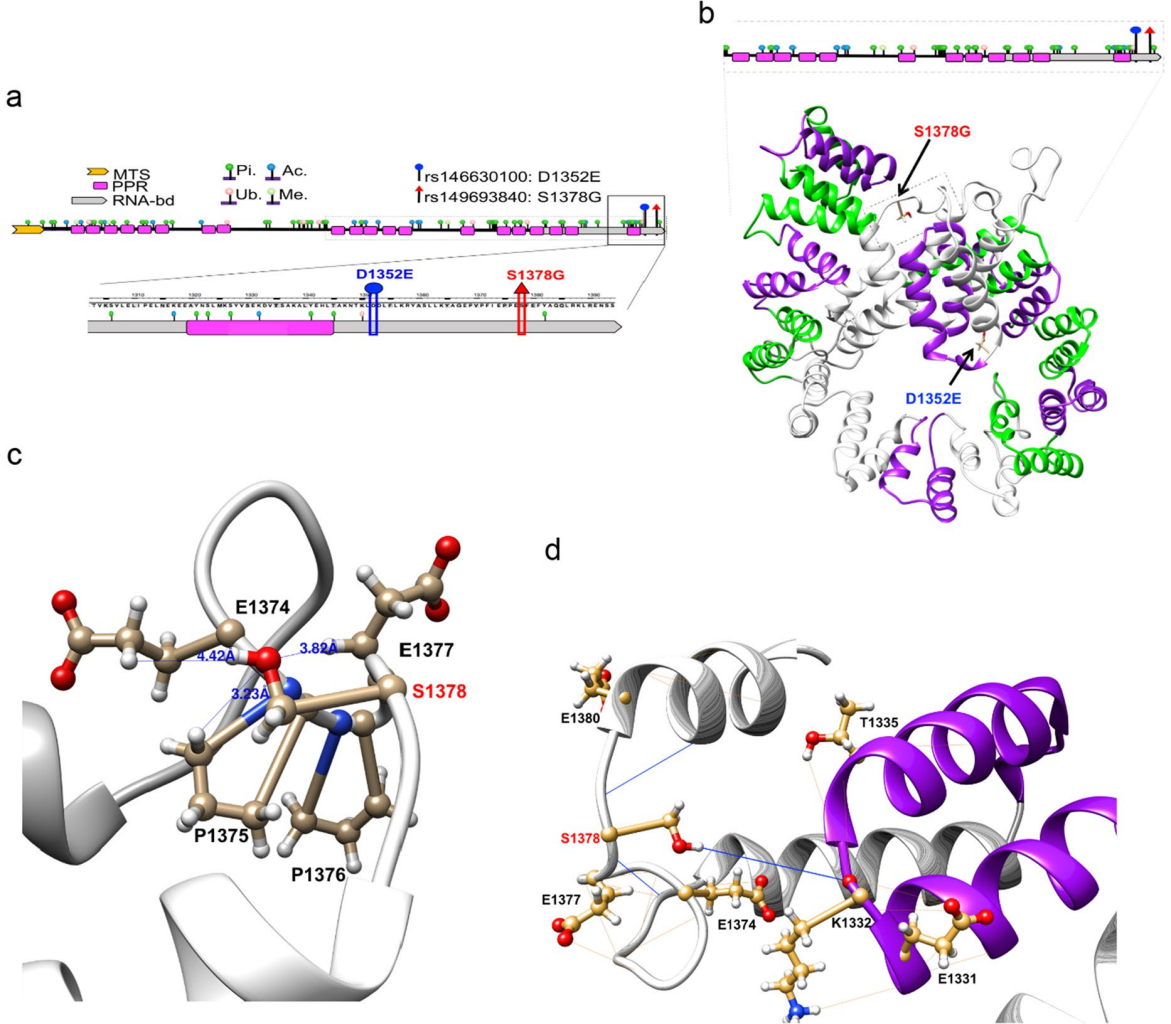
Longevity-associated variants in a proposed tertiary structure of LRPPRC. **a** 2D LRPPRC protein schematic showing the location of the N-terminal mitochondrial targeting signal (MTS), pentatricopeptide repeats (PPR), C-terminal RNA-binding domain (RNA-bd), and post-translational modification sites: phosphorylation (Pi), ubiquitinylation (Ub.), acetylation (Ac.), and methylation (Me), and longevity-associated variants D1352E and S1378G. Black box outlines the region magnified. Gray box with dashed lines approximates the portion of LRPPRC modeled by the homology structure in **b**. **b** 3D homology model with predicted PPR H–L-H motifs (green and purple) and the locations of amino acids altered by longevity-associated variants (black arrows). Gray box with dashed lines around S1378G approximates the region magnified in **c**. **c** Magnification shows loop region with distances between the oxygen atom of Ser1378 R-group with closest neighboring hydrogen atoms from Glu1374, Pro1375, and Glu1377. Distance labeled in blue and shown in angstroms (Å), distances > 5 Å omitted. **d** Potential hydrogen-bonding interactions between S1378 with the surrounding loop region and terminal PPR (helix-loop-helix motif in purple) motif are shown. Thick blue line—hydrogen bonds from Serine1378; thin orange lines—other hydrogen bonds from indicated residues

Of our highest interests are two missense variants in *LRPPRC*. These variants are very rare and only detected in centenarians (MAFs < 0.005) but not in controls or any other populations (MAF = 0) (Tables S7 and S8). rs149693840 (S1378G) is predicted to be deleterious (Table S8), while rs146630100 (D1352E) is neutral. Neither of the affected amino acids maps to positions known or predicted to undergo post-translational modification; however, a lysine ubiquitinylation site is just 2 residues upstream of D1352 and downstream of S1378, a tyrosine phosphorylation site is 4 residues away (Fig. 6a). The crystal structure of LRPPRC has never been resolved, and as of yet there is no prediction on its 3D structure.

Using publicly available protein modeling tools, we built a partial 3D homology model (“Methods” section) to visualize the location of these LAVs and their potential interactions with other residues in the tertiary structure of the C-terminus of LRPPRC (Fig. 6b-d). D1352 is located in an alpha helix, while S1378 is located in a loop that is between two alpha helices (helix-loop-helix (H-L-H) motif) (Fig. 6b, c). The closest residues to S1378 include multiple Pro and Glu residues (Fig. 6c)—P1375, P1376, E1374, E1377, and E1380—suggesting that the homology model accurately predicts loop regions as Pro residues often act as loop stabilizers due to their rigidity [77]. Moreover, loops in folded protein structures are often associated with functional domains at the surface of the protein, such as protein–protein interaction regions, especially highly ordered loops [78, 79]. The negatively charged Glu residues could participate in electrostatic interactions with other molecules and the surrounding solution; this might suggest that this is a very dynamic loop since negative charges from Glu might change the loop angle or shape to interact less with the slightly basic environment of the mitochondrial matrix where LRPPRC action takes place. Serine’s R-group contains a slightly polar carboxyl group with hydrogen-bonding capacity and we detected a potential hydrogen bond from S1378 to the backbone at residue T1322 located in the terminal H-L-H PPR motif that is involved in RNA binding (Fig. 6d). This could be important in altering the angle or position of the RNA-binding PPR or other residues and structures that are nearby in the folded tertiary structure. However, the Gly R-group is a hydrogen atom that lacks the electrostatic potential to participate in hydrogen bonding, suggesting that a substitution from S → G at residue 1378 has the potential to alter the structure and/or biochemistry of the C-terminal RNA-binding domain in LRPPRC. *LRPPRC* has yet not been directly investigated in the context of aging and longevity, making it a novel target for our future study.

## Discussion

By combining a targeted candidate sequencing approach with integrative analysis of potentially functional centenarian-enriched, rare variants, we discovered 35 high-priority functional variants (LAVs) and 75 mitonuclear genes (LAGs) in multiple mitochondrial pathways that are associated with longevity (*p* < 0.05). Our data support the hypothesis that rare variants are likely functional and enriched in individuals exhibiting the rare phenotype of extreme longevity.

Given the rarity of these variants in the rare population of centenarians, it is highly challenging, if not impossible, to establish the statistical significance of the longevity association. However, focusing on the well-established conserved pathways of aging, we have identified and characterized longevity-associated functional rare coding variants in *IGF1R* [37, 38] and functional rare non-coding variants in *NFKBIA* [39], *CLU* [39], and *PRKCH* [39] genes by using cell models. Our results demonstrated parallel mechanisms between humans and model organisms, increasing our confidence that longevity-associated genes/pathways found in the conserved pathways may be relevant to human longevity.

We identified nutrient sensing through MTOR, ketone body metabolism, calcium signaling, the urea cycle, 1-carbon metabolism (1C), and mitochondrial translation as potential key targets for the investigation of the mitochondrial mechanisms of human longevity. We targeted two rare variants in *LRPPRC* gene, rs149693840 (S1378G) and rs146630100 (D1352E) that are located in the C-terminal RNA-binding domain of the mitochondrial translation regulator [80]. These variants are adjacent to residues that undergo post-translational modification, suggesting that altering these amino acids has the potential to impact protein–protein interactions and signaling. The S1378G variant has the potential to alter electrostatic interactions that stabilize a loop structure and the terminal H-L-H PPR motif, involved in the RNA-binding function of LRPPRC. Autosomal recessive missense mutations in *LRPPRC* cause the French-Canadian Type of Leigh Syndrome (LSFC), a rare mitochondrial disease-causing cytochrome c oxidase (COX, complex IV (CIV)) deficiency, which results in severe neurological, muscular, and kidney pathology, lactic acidosis, and premature death [81–83]. Loss of function of *LRPPRC* in worms and human cell lines disrupts ETC stoichiometry, especially at CIV, leading to mitochondrial protein imbalance, upregulated mtUPR, altered ATP/ADP ratio, and altered mitochondrial dynamics [84, 85]. Given previous enigmatic work on ETC dysfunction in model organism longevity [14, 74, 86, 87], the importance of mitochondrial translation and proteostasis in mediating several mechanisms of longevity and aging in model organisms, and the biochemical importance of COX in regulating the rate of respiration [88], we believe these LAVs could be interesting targets for future investigation.

We identified *MFN2* as a LAG in two SKATs and validated a rare enriched stop gain LAV (G548*) in this gene that is predicted to be deleterious by CADD and located at a highly evolutionarily conserved residue. MFN2 is a positive regulator of mitochondrial dynamics and morphology and tethers the mitochondria and ER membranes to form mitochondrial-associated membranes (MAMs) that regulate lipid and Ca^2+^ exchange at the ER, and regulate ubquinone (UQ) synthesis [89–91]. Given that complete knockout (KO) of *MFN2* is embryonic lethal in mice and loss of function mutations in humans lead to the severe mitochondrial disease Charcot Marie Tooth Type 2A (CMT2A) [92], the heterozygous carriers of this variant as detected in centenarians may minimally reduce protein abundance or function. Alternatively, the truncated protein only interrupts a short portion of the cytoplasmic domain but does not completely abolish the remaining C-terminal region of the protein which would render it completely non-functional [93]. Moreover, it could be that this *MFN2* variant partially alters localization of MFN2 [94], or interactions with other proteins, including PINK1 and PARKIN which, as mentioned above, are important mediators in the etiology of dementia, including Alzheimer’s disease (AD) and PD [95], or the variant may alter UQ biosynthesis or export to extramitochondrial endomembrane systems [89, 96].

A rare frameshift in *MTOR* (Y2396Lfs*29) enriched in centenarians has the strong potential to impair MTOR function. This *MTOR* frameshift was the only significant coding variant identified in the *MTOR* gene and the only significant frameshift variant in the sequencing data, suggesting that this variant is extremely unique. Given the background and established role of MTOR on aging and lifespan determination as well as on mitochondrial biogenesis and metabolism [97–99], it is intriguing to speculate that this frameshift produces some functional effect on MTOR protein abundance or signaling. We hypothesize that this variant may reduce mTOR-mediated nutrient sensing and signaling, thereby increasing lifespan and healthspan through multiple downstream mechanisms, including enhanced autophagy, improved mitochondrial function, and altered metabolism, as has been shown in model organisms and cultured human cells [10, 100–103].

The functional CPS1 variant (rs1047891; T1406N) occurs in the allosteric binding domain of the protein. Studies suggest it confers protection from coronary artery disease (CAD) risk in females specifically and metabolic disease (MD) through control of weight gain, suggesting that this variant could be an example of a sexually dimorphic variant in longevity [104–109]. CPS1 is an enzymatic hub in mitochondrial metabolism linked to caloric restriction, SIRT5, the TCA cycle, 1C metabolism, and NO production, and its primary role is in detoxification of NH4^+^ by the urea cycle which is increased under nutrient deprivation stress when protein catabolism increases [105, 110–112].

A functional investigation of these variants, either in vitro or in vivo, may help resolve the biological mechanisms behind complex phenotypes like longevity across species. Future studies of these rare variants with knock-in models in human cell lines or mammalian model organisms are critical to providing the functional evidence that validates these in silico results. In studying the role of mitochondria in human aging and longevity, it is critical to consider how cellular metabolic, redox, and oxygen environments might modify the phenotype or how polymorphic longevity-associated pathways might change in response to different cellular stress conditions.

It is known that complex phenotypes are determined by multiple alleles functioning in combination (i.e., haplotypes), which should be extended to trans-genomic haplotypes and interactions between mtDNA-nDNA in future research [113–115]. With future developments in the integration of genomics, epigenomics, transcriptomics, proteomics, and metabolomics (-omics) data, there is greater hope that functional genetic variation in any pathway can be investigated for any phenotype, and in humans, on a more holistic system-biology level [1, 2, 116]. This kind of work will also help researchers plan for focused data-driven experiments aimed at explaining genotype–to–phenotype mechanisms.

Our results suggest new hypotheses and support existing hypotheses that may help guide future experiments aimed at linking important functions of mitonuclear genes to human longevity. Future studies should explore these hypotheses in functional biological models.

## Supplementary Information

Below is the link to the electronic supplementary material.

Supplementary file1 (DOCX 622 KB)

## Data Availability

Not applicable.
