# Supplementary material for: High-throughput sequencing analysis of nuclear-encoded mitochondrial genes reveals a genetic signature of human longevity
Source: GeroScience. 2022 Aug 10;45(1):311–30. doi: 10.1007/s11357-022-00634-z (PMC9886794; doi:10.1007/s11357-022-00634-z)
Supplement: Supplementary file 1 — Supplementary file1 (DOCX 622 KB) [file 11357_2022_634_MOESM1_ESM.docx]

a

**
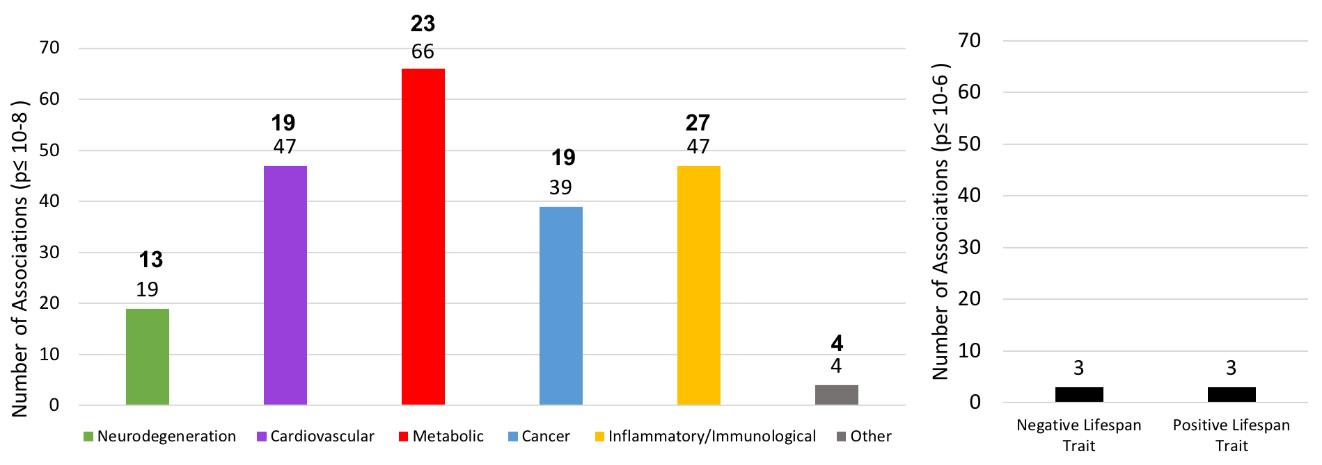
**
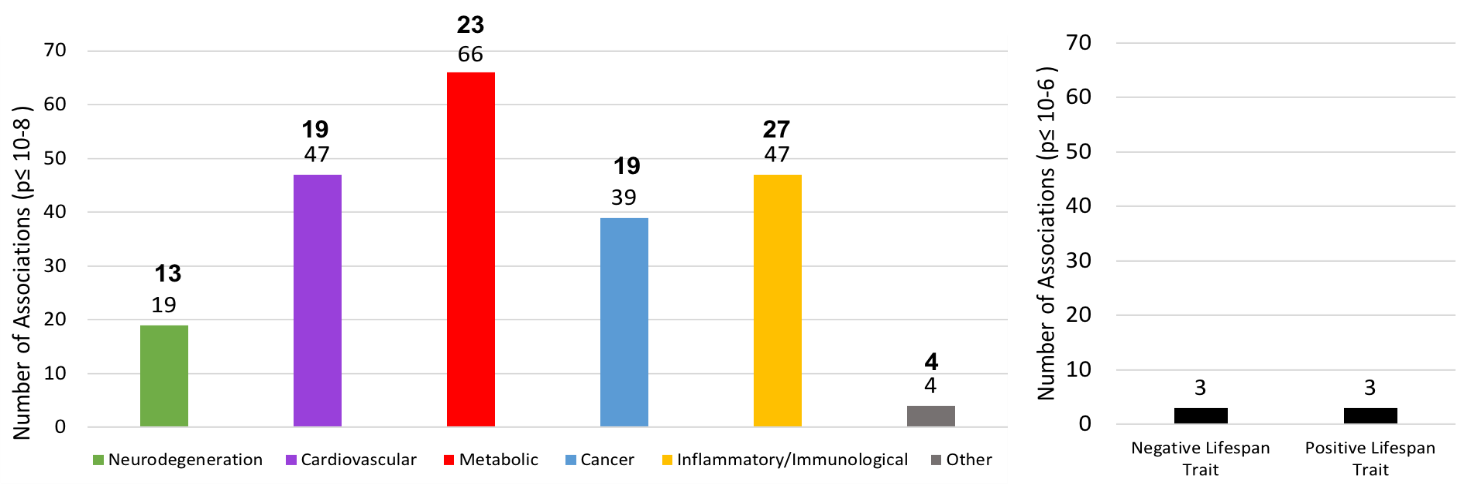


b


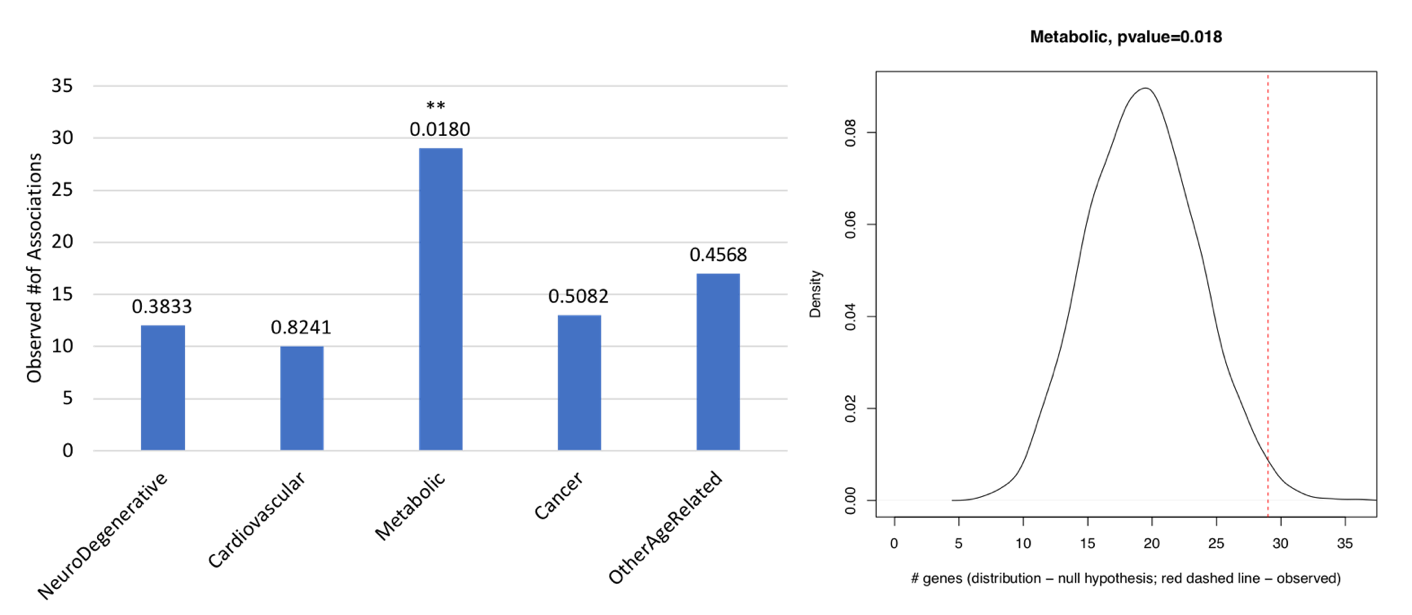


c

d

**
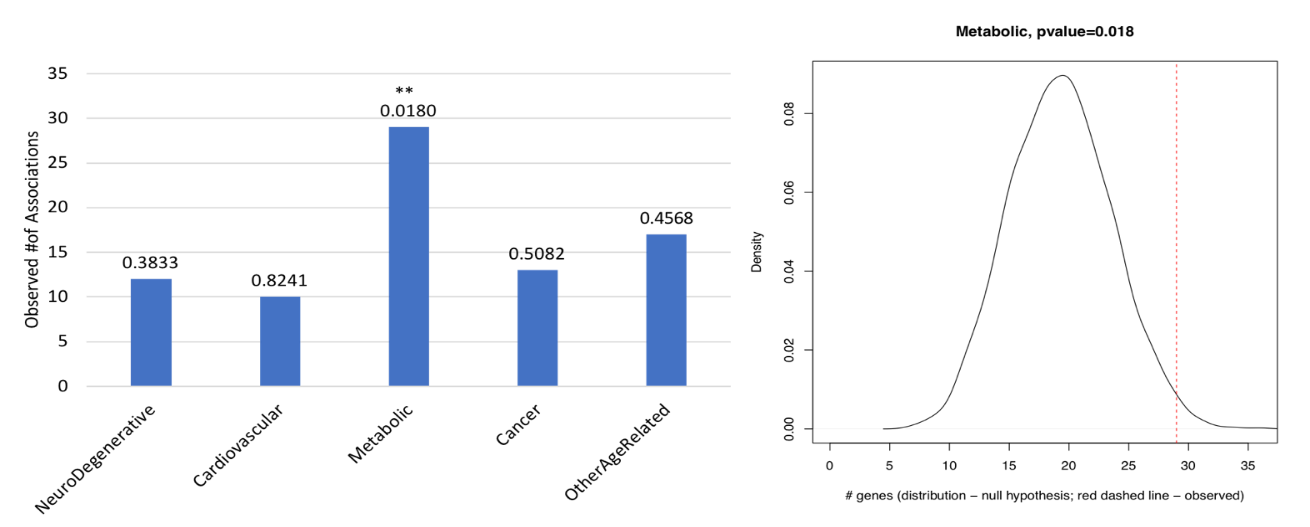
**

**Neurodegenerative**

**Cardiovascular**

**Cancer**

**Metabolic**

**Other age-related**

**Fig. S1 Association with age-related diseases of mitonuclear candidate gene common variants in GWAS.** **a.** Reported number of associations at the genome-wide significance cutoff of 10^-8^ was used for age-related diseases and biomarkers. Non-bold numbers above bars indicate number of associations with the disease category, and bold numbers above bars indicate the number of genes with those associations. **b.** Mitonuclear candidate associations with aging, lifespan, and longevity in GWAS. Reported number of associations at a less stringent genome-wide significance cutoff of 10^-6^ was used for lifespan-specific traits. Number above the bar indicates number of associations. **c.** Significant enrichment of mitonuclear candidate genes in GWAS of metabolic disease. The red dotted line indicates the significant enrichment of the mitonuclear candidate gene set within the distribution of genes associated with metabolic disease in GWAS, and p-value (0.018) as determined by the EARDiG algorithm. **d.** Enrichment of the mitonuclear candidate gene set compared to background sets of genes associated with different age-related diseases in GWAS (p≤1x10^-8^), p-value of enrichment is reported at the top of each bar. Observed number of mitonuclear candidate gene associations per GWAS age-related disease group was obtained by EARDiG and replotted to generate a bar graph for comparison.

a

b

**Fig. S2 Distribution of variants in controls and cases including or excluding singletons. a.** Distribution of variants in controls and cases, including singletons. Percentages of different types of genetic variations in controls and centenarians are only negligibly different by 1% for certain types of variants (*i.e.,* 1% more intronic, less 3UTR, and less InDels in cases compared to controls). **b.** Distribution of variants in controls and cases excluding singletons. Distribution and percentages of different types of genetic variations by variant counts in controls and centenarians are negligibly different by only 1% for certain types of variants (*i.e.,* 1% less sVs and more Fs).

a

b

**Fig. S3 Distribution of mitonuclear gene variants in controls and centenarians including or excluding singletons.**  **a.** Distribution of mitonuclear gene variants in controls. Distribution of different types of genetic variations in controls changes only negligibly after removal of singleton variants (*e.g.,* 5% more sVs and 4% less nsVs after excluding singletons). **b.** Distribution of mitonuclear gene variants in centenarians. Distribution and percentages of different types of genetic variations by variant counts in centenarians changes only negligibly after removal of singleton variants (*e.g.,* 3% more sVs and 3% less nsVs after excluding singletons).

**Table S1. Ontology terms and functions in mitonuclear candidate gene categories.**

| **Category** | **Ontology terms and biological processes** |
| --- | --- |
| Lipid Metabolism | Catabolism, anabolism, and transporters of fatty acids, phospholipids, glycerolipids, triacylglycerides, sphingolipids, steroid hormones, cholesterol, bile-acid, and ketones |
| Oxidative Phosphorylation | Respiratory gas exchange, cellular respiration, ATP synthesis via chemiosmotic coupling, electron transport chain - subunits, assembly proteins, and succinate dehydrogenase^$^ (CII and TCA cycle), Coenzyme Q/ubiquinone/ubiquinol metabolism |
| Carbohydrate Metabolism | Catabolism, anabolism, and transporters of glycolysis, gluconeogenesis, TCA cycle, and pentose phosphate pathway  Galactose, fructose, mannose, glyoxylate, pyruvate, isocitrate, 2-oxoglutarate |
| Metabolism – Other | Amino acid, polyamines, creatine, vitamin, nucleotides^$^, nitrogenous compounds, urea, and xenobiotics |
| Redox and Calcium Signaling | Iron-sulfur cluster metabolism, hemostasis, heme and porphyrin metabolism, angiogenesis  Ion channels and transporters for calcium signaling and homeostasis  Oxidative stress responses^$^ - ROS metabolism, hypoxia, oxidative DNA demethylation^$^, glutathione, lipoxygenase, regulation of oxidation, inflammatory and immune response^$^ |
| Structural Dynamics and Stress Responses^$^ | Fusion-fission, mitochondrial network organization, morphology, cristae maintenance and MICOS complex  Mitochondrial transport on microtubules/anterograde transport  Degradation – autophagy, apoptosis^$^ – cytochrome c release and mitochondrial permeability transition pore formation  Inflammatory and immune signaling^$^, senescence^$^, cell survival^$^, morphogenesis, development, proliferation^$^ and signaling pathways (MTOR, PARP, Interleukin, etc.)^$^ |
| Protein Expression and Stasis^$^ | Translation: ribosomal proteins, tRNA processing and modification, mt-mRNA and peptide processing and maturation  Proteostasis and protein quality control: chaperones^$^, unfolded protein response, protein modification and processing, protein complex assembly^$^  Protein targeting to the mitochondria – import and export of mitochondrial peptides |
| Gene Expression and Cell Cycle | MtDNA and mt-nDNA expression: transcription, replication, synthesis and regulation required for biogenesis and proliferation of mitochondria  DNA damage response and repair^$^  inflammatory and immune response^$,^ Cell cycle^$^, cell growth^$^, differentiation^$^, meiosis, and mitosis, senescence^$^, Apoptosis^$^ |

^$^Functions and terms that overlap among some categories. The proteins with these ontology terms are more difficult to confidently place in a single category because they often serve context dependent functions and have moonlighting abilities that make their precise and discrete functional categorization an impossible task.

Table S2. Categorized mitonuclear candidate gene list.

| **Entrez Gene ID** | **Gene Symbol** | **Category** |
| --- | --- | --- |
| 30 | ACAA1 | Lipid Metabolism |
| 31 | ACACA | Lipid Metabolism - Ketone/ Vitamin Metabolism |
| 32 | ACACB | Lipid Metabolism |
| 33 | ACADL | Lipid Metabolism- Beta-Oxidation |
| 34 | ACADM | Lipid Metabolism- Beta-Oxidation |
| 35 | ACADS | Lipid Metabolism |
| 36 | ACADSB | Lipid Metabolism/ Amino Acid Metabolism |
| 37 | ACADVL | Lipid Metabolism/ Proteostasis - UPR |
| 38 | ACAT1 | Lipid Metabolism - Ketones |
| 39 | ACAT2 | Lipid Metabolism |
| 1374 | CPT1A | Lipid Metabolism |
| 1891 | ECH1 | Lipid Metabolism |
| 427 | ASAH1 | Lipid Metabolism - Sphingolipid |
| 622 | BDH1 | Lipid Metabolism - Ketone Metabolism |
| 1119 | CHKA | Lipid Metabolism |
| 1120 | CHKB | Lipid Metabolism |
| 1962 | EHHADH | Lipid Metabolism |
| 1375 | CPT1B | Lipid Metabolism- Beta-Oxidation, Lipid Transport |
| 1376 | CPT2 | Lipid Metabolism |
| 1583 | CYP11A1 | Lipid Metabolism -Steroid Hormone/ Redox |
| 1584 | CYP11B1 | Lipid Metabolism -Steroid Hormone/ Redox |
| 1585 | CYP11B2 | Lipid Metabolism- Hormone and Cholesterol |
| 2181 | ACSL3 | Lipid Metabolism |
| 3032 | HADHB | Lipid Metabolism |
| 5444 | PON1 | Lipid Metabolism |
| 1632 | ECI | Lipid Metabolism |
| 1666 | DECR1 | Lipid Metabolism |
| 1717 | DHCR7 | Lipid Metabolism- Cholesterol |
| 6296 | ACSM3 | Lipid Metabolism |
| 1892 | ECHS1 | Lipid Metabolism |
| 6576 | SLC25A1 | Lipid Metabolism |
| 2167 | FABP4 | Lipid Metabolism |
| 2180 | ACSL1 | Lipid Metabolism |
| 1593 | CYP27A1 | Lipid Metabolism - Bile Acids, Sterols/Redox |
| 2182 | ACSL4 | Lipid Metabolism |
| 2194 | FASN | Lipid Metabolism/ Vitamin Metabolism |
| 2531 | KDSR | Lipid Metabolism - Sphingolipid |
| 2581 | GALC | Lipid Metabolism |
| 2710 | GK | Lipid Metabolism / Carbohydrate Metabolism |
| 4047 | LSS | Lipid Metabolism - Cholesterol |
| 3030 | HADHA | Lipid Metabolism- Beta-Oxidation |
| 221 | ALDH3B1 | Lipid Metabolism - Sphingolipid/Redox |
| 3033 | HADH | Lipid Metabolism- Beta-Oxidation |
| 1589 | CYP21A2 | Lipid Metabolism - Steroids/Redox |
| 3156 | HMGCR | Lipid Metabolism- Cholesterol |
| 3157 | HMGCS1 | Lipid Metabolism- Cholesterol and Ketones |
| 6721 | SREBF2 | Lipid Metabolism- Cholesterol/Gene Expression |
| 3990 | LIPC | Lipid Metabolism- Cholesterol |
| 6307 | MSMO1 | Lipid Metabolism- Cholesterol/Redox |
| 6309 | SC5DL | Lipid Metabolism- Cholesterol/Redox |
| 5095 | PCCA | Lipid Metabolism / Amino Acid and Vitamin Metabolism |
| 5096 | PCCB | Lipid Metabolism / Amino Acid and Vitamin Metabolism |
| 9524 | TECR | Lipid Metabolism |
| 5297 | PI4KA | Lipid Metabolism - Phospholipids |
| 5298 | PI4KB | Lipid Metabolism |
| 5305 | PI4K2A | Lipid Metabolism- Phospholipids / Autophagy |
| 5406 | PNLIP | Lipid Metabolism |
| 51703 | ACSL5 | Lipid Metabolism |
| 5564 | PRKAB1 | Moonlighting - Lipid Metabolism/AMPK - Metabolic Integration/Biogenesis, Stress Response, Signaling, Gene Expression |
| 5571 | PRKAG1 | Moonlighting - Lipid Metabolism/AMPK - Metabolic Integration/ Biogenesis, Stress Response, Signaling, Gene Expression |
| 5833 | PCYT2 | Lipid Metabolism |
| 55856 | ACOT13 | Lipid Metabolism |
| 3155 | HMGCL | Lipid Metabolism- Ketones |
| 65985 | AACS | Lipid Metabolism- Ketones |
| 3158 | HMGCS2 | Lipid Metabolism- Ketones and Cholesterol |
| 6720 | SREBF1 | Lipid Metabolism |
| 3992 | FADS1 | Lipid Metabolism- Phospholipids |
| 5130 | PCYT1A | Lipid Metabolism- Phospholipids |
| 6901 | TAZ | Lipid Metabolism |
| 8309 | ACOX2 | Lipid Metabolism - Bile Acid Synthesis/ Redox |
| 8310 | ACOX3 | Lipid Metabolism |
| 8396 | PIP4K2B | Lipid Metabolism- Phospholipids / Autophagy |
| 55300 | PI4K2B | Lipid Metabolism- Phospholipids |
| 56994 | CHPT1 | Lipid Metabolism- Phospholipids |
| 55304 | SPTLC3 | Lipid Metabolism- Sphingolipid |
| 56624 | ASAH2 | Lipid Metabolism- Sphingolipid |
| 9415 | FADS2 | Lipid Metabolism |
| 9468 | PCYT1B | Lipid Metabolism |
| 340485 | ACER2 | Lipid Metabolism- Sphingolipid |
| 9517 | SPTLC2 | Lipid Metabolism- Sphingolipid |
| 10400 | PEMT | Lipid Metabolism |
| 91012 | CERS5 | Lipid Metabolism- Sphingolipid |
| 6770 | STAR | Lipid Metabolism- Steroid Hormone and Cholesterol |
| 10558 | SPTLC1 | Lipid Metabolism - Sphingolipid |
| 123876 | ACSM2A | Lipid Metabolism/Amino Acid Metabolism/ Xenobiotic Detoxification |
| 10965 | ACOT2 | Lipid Metabolism |
| 10998 | SLC27A5 | Lipid Metabolism - Ketones and Bile Acid Synthesis |
| 11001 | SLC27A2 | Lipid Metabolism- Bile Acid |
| 23597 | ACOT9 | Lipid Metabolism |
| 2820 | GPD2 | Lipid Metabolism/Gluconeogenesis |
| 23761 | PISD | Lipid Metabolism - Phospholipids |
| 239 | ALOX12 | Lipid Metabolism/Redox |
| 28976 | ACAD9 | Lipid Metabolism / ETC - CI Assembly |
| 29956 | CERS2 | Lipid Metabolism - Sphingolipid |
| 1595 | CYP51A1 | Lipid Metabolism/Redox |
| 8513 | LIPF | Lipid Metabolism/Redox |
| 50814 | NSDHL | Lipid Metabolism - Cholesterol/Redox |
| 8611 | PPAP2A | Lipid Metabolism |
| 8612 | PPAP2C | Lipid Metabolism |
| 8613 | PPAP2B | Lipid Metabolism |
| 10449 | ACAA2 | Lipid Metabolism |
| 55326 | AGPAT5 | Lipid Metabolism |
| 55500 | ETNK1 | Lipid Metabolism |
| 10455 | ECI2 | Lipid Metabolism |
| 23659 | PLA2G15 | Lipid Metabolism |
| 55902 | ACSS2 | Lipid Metabolism |
| 27349 | MCAT | Lipid Metabolism |
| 51102 | MECR | Lipid Metabolism |
| 57205 | ATP10D | Lipid Metabolism - Transporter |
| 57678 | GPAM | Lipid Metabolism |
| 51205 | ACP6 | Lipid Metabolism |
| 54995 | OXSM | Lipid Metabolism |
| 55825 | PECR | Lipid Metabolism |
| 84129 | ACAD11 | Lipid Metabolism |
| 84188 | FAR1 | Lipid Metabolism |
| 79746 | ECHDC3 | Lipid Metabolism |
| 84947 | SERAC1 | Lipid Metabolism |
| 85465 | EPT1 | Lipid Metabolism |
| 116285 | ACSM1 | Lipid Metabolism |
| 126129 | CPT1C | Lipid Metabolism |
| 125981 | ACER1 | Lipid Metabolism |
| 165679 | SPTSSB | Lipid Metabolism |
| 171546 | SPTSSA | Lipid Metabolism |
| 220832 | FABP5L3 | Lipid Metabolism |
| 347516 | DGAT2L6 | Lipid Metabolism |
| 653308 | ASAH2B | Lipid Metabolism |
| 5728 | PTEN | Moonlighting - Stress Response/Lipid Metabolism - Phospholipid, PIP/Signaling - Cancer Metabolism, |
| 10577 | NPC2 | Lipid Metabolism - Transporter |
| 348158 | ACSM2B | Lipid Metabolism/ Amino Acid Metabolism |
| 80724 | ACAD10 | Lipid Metabolism |
| 1329 | COX5B | Oxidative Phosphorylation |
| 1337 | COX6A1 | Oxidative Phosphorylation |
| 1340 | COX6B1 | Oxidative Phosphorylation |
| 1349 | COX7B | Oxidative Phosphorylation |
| 1355 | COX15 | Oxidative Phosphorylation |
| 1537 | CYC1 | Oxidative Phosphorylation |
| 2108 | ETFA | Oxidative Phosphorylation |
| 2109 | ETFB | Oxidative Phosphorylation |
| 4697 | NDUFA4 | Oxidative Phosphorylation |
| 4715 | NDUFB9 | Oxidative Phosphorylation |
| 4722 | NDUFS3 | Oxidative Phosphorylation |
| 4728 | NDUFS8 | Oxidative Phosphorylation |
| 7381 | UQCRB | Oxidative Phosphorylation |
| 7384 | UQCRC1 | Oxidative Phosphorylation |
| 7385 | UQCRC2 | Oxidative Phosphorylation |
| 7386 | UQCRFS1 | Oxidative Phosphorylation |
| 7388 | UQCRH | Oxidative Phosphorylation |
| 1345 | COX6C | Oxidative Phosphorylation / Gene Expression |
| 9167 | COX7A2L | Oxidative Phosphorylation |
| 10063 | COX17 | Oxidative Phosphorylation |
| 27089 | UQCRQ | Oxidative Phosphorylation |
| 126328 | NDUFA11 | Oxidative Phosphorylation |
| 708396 | UQCR | Oxidative Phosphorylation |
| 4717 | NDUFC1 | Oxidative Phosphorylation |
| 4704 | NDUFA9 | Oxidative Phosphorylation |
| 4696 | NDUFA3 | Oxidative Phosphorylation - ETC - CI |
| 4706 | NDUFAB1 | Oxidative Phosphorylation - ETC - CI |
| 4713 | NDUFB7 | Oxidative Phosphorylation - ETC - CI |
| 4714 | NDUFB8 | Oxidative Phosphorylation - ETC - CI |
| 2110 | ETFDH | Oxidative Phosphorylation/ Redox |
| 51103 | NDUFAF1 | Oxidative Phosphorylation - ETC - CI |
| 100173591 | UQCR10 | Oxidative Phosphorylation - ETC - CIII |
| 1327 | COX4I1 | Oxidative Phosphorylation - ETC - CIV |
| 6391 | SDHC | Oxidative Phosphorylation / TCA Cycle - Ubiquinone/ Redox |
| 51204 | CCDC44 | Oxidative Phosphorylation / TCA Cycle/ Gene Expression |
| 1350 | COX7C | Oxidative Phosphorylation- ETC |
| 1353 | COX11 | Oxidative Phosphorylation - ETC - CIV |
| 4702 | NDUFA8 | Oxidative Phosphorylation - CI / TCA Cycle |
| 4723 | NDUFV1 | Oxidative Phosphorylation - ETC - CI |
| 4705 | NDUFA10 | Oxidative Phosphorylation - ETC - CI |
| 4694 | NDUFA1 | Oxidative Phosphorylation - ETC - CI |
| 4695 | NDUFA2 | Oxidative Phosphorylation - ETC - CI |
| 4698 | NDUFA5 | Oxidative Phosphorylation - ETC - CI |
| 4710 | NDUFB4 | Oxidative Phosphorylation - ETC - CI/ TCA Cycle |
| 4700 | NDUFA6 | Oxidative Phosphorylation - ETC - CI |
| 4701 | NDUFA7 | Oxidative Phosphorylation - ETC - CI |
| 4708 | NDUFB2 | Oxidative Phosphorylation - ETC - CI |
| 4709 | NDUFB3 | Oxidative Phosphorylation - ETC - CI |
| 4711 | NDUFB5 | Oxidative Phosphorylation - ETC - CI |
| 4716 | NDUFB10 | Oxidative Phosphorylation - ETC - CI |
| 4712 | NDUFB6 | Oxidative Phosphorylation - ETC - CI |
| 4718 | NDUFC2 | Oxidative Phosphorylation - ETC - CI |
| 4719 | NDUFS1 | Oxidative Phosphorylation - ETC - CI/ Apoptosis |
| 4720 | NDUFS2 | Oxidative Phosphorylation - ETC - CI |
| 4724 | NDUFS4 | Oxidative Phosphorylation - ETC - CI |
| 4725 | NDUFS5 | Oxidative Phosphorylation - ETC - CI |
| 4726 | NDUFS6 | Oxidative Phosphorylation - ETC - CI |
| 4729 | NDUFV2 | Oxidative Phosphorylation - ETC - CI |
| 51300 | C3orf1 | Oxidative Phosphorylation - ETC - CI |
| 1351 | COX8A | Oxidative Phosphorylation - ETC - CIV |
| 285521 | COX18 | Oxidative Phosphorylation - ETC - CIV |
| 4731 | NDUFV3 | Oxidative Phosphorylation - ETC - CI |
| 6341 | SCO1 | Oxidative Phosphorylation/Gene Expression |
| 6389 | SDHA | Oxidative Phosphorylation - ETC - CII / TCA Cycle |
| 1352 | COX10 | Oxidative Phosphorylation - ETC - CIV/ Heme and Porphyrin Metabolism |
| 509 | ATP5C1 | Oxidative Phosphorylation |
| 498 | ATP5A1 | Oxidative Phosphorylation |
| 6834 | SURF1 | Oxidative Phosphorylation - CIV Assembly |
| 506 | ATP5B | Oxidative Phosphorylation |
| 513 | ATP5D | Oxidative Phosphorylation |
| 514 | ATP5E | Oxidative Phosphorylation |
| 515 | ATP5F1 | Oxidative Phosphorylation |
| 516 | ATP5G1 | Oxidative Phosphorylation |
| 517 | ATP5G2 | Oxidative Phosphorylation |
| 9377 | COX5A | Oxidative Phosphorylation - CIV |
| 518 | ATP5G3 | Oxidative Phosphorylation |
| 521 | ATP5I | Oxidative Phosphorylation |
| 522 | ATP5J | Oxidative Phosphorylation |
| 539 | ATP5O | Oxidative Phosphorylation |
| 1339 | COX6A2 | Oxidative Phosphorylation |
| 1346 | COX7A1 | Oxidative Phosphorylation |
| 1347 | COX7A2 | Oxidative Phosphorylation |
| 9551 | ATP5J2 | Oxidative Phosphorylation |
| 10476 | ATP5H | Oxidative Phosphorylation |
| 10632 | ATP5L | Oxidative Phosphorylation |
| 51079 | NDUFA13 | Oxidative Phosphorylation - ETC - CI/ Redox/Proteostasis/Apoptosis |
| 27109 | ATP5S | Oxidative Phosphorylation |
| 51117 | COQ4 | Oxidative Phosphorylation |
| 54968 | TMEM70 | Oxidative Phosphorylation |
| 55245 | UQCC | Oxidative Phosphorylation |
| 54205 | CYCS | Oxidative Phosphorylation - ETC /Apoptosis/Biogenesis/Stress Response |
| 54539 | NDUFB11 | Oxidative Phosphorylation - ETC - CI/ Redox |
| 55967 | NDUFA12 | Oxidative Phosphorylation |
| 56997 | ADCK3 | Oxidative Phosphorylation |
| 64756 | ATPAF1 | Oxidative Phosphorylation |
| 79085 | SLC25A23 | Oxidative Phosphorylation |
| 84300 | C6orf125 | Oxidative Phosphorylation |
| 84701 | COX4I2 | Oxidative Phosphorylation |
| 90624 | LYRM7 | Oxidative Phosphorylation |
| 91647 | ATPAF2 | Oxidative Phosphorylation |
| 93974 | ATPIF1 | Oxidative Phosphorylation |
| 125965 | COX6B2 | Oxidative Phosphorylation |
| 144363 | LYRM5 | Oxidative Phosphorylation |
| 341947 | COX8C | Oxidative Phosphorylation |
| 91942 | NDUFA12L | Oxidative Phosphorylation - ETC - CI Assembly |
| 100131801 | PET100 | Oxidative Phosphorylation |
| 6390 | SDHB | Oxidative Phosphorylation / TCA Cycle - Pyruvate Metabolism |
| 6392 | SDHD | Oxidative Phosphorylation / TCA Cycle - Pyruvate Metabolism |
| 10229 | COQ7 | Oxidative Phosphorylation - Ubiquinone/Lipid Metabolism/Redox |
| 23590 | PDSS1 | Oxidative Phosphorylation - Ubiquinone/Lipid Metabolism/Redox |
| 27235 | COQ2 | Oxidative Phosphorylation - Ubiquinone/Lipid Metabolism/Redox |
| 374291 | NDUFS7 | Oxidative Phosphorylation - ETC - CI |
| 51004 | COQ6 | Oxidative Phosphorylation - Ubiquinone/Lipid Metabolism/Redox |
| 57107 | PDSS2 | Oxidative Phosphorylation - Ubiquinone/Lipid Metabolism/Redox |
| 54949 | SDHAF2 | Oxidative Phosphorylation / TCA Cycle |
| 644096 | SDHAF1 | Oxidative Phosphorylation / TCA Cycle |
| 50 | ACO2 | TCA Cycle |
| 226 | ALDOA | Carbohydrate Metabolism- Glycolysis and Gluconeogenesis |
| 229 | ALDOB | Carbohydrate Metabolism- Glycolysis and Gluconeogenesis |
| 230 | ALDOC | Carbohydrate Metabolism- Glycolysis/Gluconeogenesis |
| 1431 | CS | Carbohydrate Metabolism- TCA Cycle |
| 1738 | DLD | TCA Cycle & Oxphos- TCA - NADH To Ubiquinone |
| 1743 | DLST | Carbohydrate Metabolism and Oxphos- TCA |
| 2023 | ENO1 | Carbohydrate Metabolism- Glycolysis and Gluconeogenesis |
| 2026 | ENO2 | Carbohydrate Metabolism- Glucose - Glycolysis |
| 2027 | ENO3 | Carbohydrate Metabolism- Glycolysis and Gluconeogenesis |
| 2203 | FBP1 | Carbohydrate Metabolism- Glucose - Glycolysis and Gluconeogenesis |
| 2271 | FH | Carbohydrate Metabolism- TCA Cycle |
| 2582 | GALE | Carbohydrate Metabolism- Galactose |
| 2584 | GALK1 | Carbohydrate Metabolism- Galactose |
| 2592 | GALT | Carbohydrate Metabolism- Galactose |
| 2597 | GAPDH | Carbohydrate Metabolism- Glycolysis |
| 2646 | GCK | Carbohydrate Metabolism |
| 2806 | GOT2 | Carbohydrate Metabolism - Gluconeogenesis and Amino Acids |
| 2821 | GPI | Carbohydrate Metabolism - Glycolysis and Glucose |
| 3098 | HK1 | Carbohydrate Metabolism and Transport - Glycolysis |
| 3099 | HK2 | Carbohydrate Metabolism- Glycolysis |
| 3417 | IDH1 | TCA/Redox/Lipid Metabolism |
| 3418 | IDH2 | Carbohydrate Metabolism - TCA |
| 3419 | IDH3A | Carbohydrate Metabolism- TCA Cycle |
| 3420 | IDH3B | TCA Cycle And ETC |
| 3421 | IDH3G | TCA Cycle/Carbohydrate Metabolism |
| 4190 | MDH1 | Carbohydrate Metabolism- Gluconeogenesis And TCA |
| 4191 | MDH2 | Carbohydrate Metabolism- TCA Cycle |
| 4199 | ME1 | Carbohydrate Metabolism - Cofactor NADPH Metabolism/ Lipid Metabolism |
| 4200 | ME2 | TCA And Carbohydrate Metabolism |
| 4967 | OGDH | Carbohydrate Metabolism- TCA Cycle |
| 5105 | PCK1 | Carbohydrate Metabolism- Glucose |
| 5106 | PCK2 | Carbohydrate Metabolism - Glycolysis and Gluconeogenesis |
| 5160 | PDHA1 | Carbohydrate Metabolism- TCA |
| 5161 | PDHA2 | Carbohydrate and TCA Metabolism |
| 5162 | PDHB | Carbohydrate Metabolism - TCA Cycle |
| 5163 | PDK1 | TCA Cycle |
| 5164 | PDK2 | Carbohydrate Metabolism - TCA Cycle and Glycolysis |
| 5166 | PDK4 | Carbohydrate Metabolism - TCA Cycle |
| 5207 | PFKFB1 | Carbohydrate Metabolism- Glycolysis/Gluconeogenesis |
| 5211 | PFKL | Carbohydrate Metabolism- Glycolysis |
| 5213 | PFKM | Carbohydrate Metabolism - Glycolysis |
| 5214 | PFKP | Carbohydrate Metabolism- Glucose - Glycolysis |
| 5223 | PGAM1 | Carbohydrate Metabolism- Glycolysis/Gluconeogenesis |
| 5224 | PGAM2 | Carbohydrate Metabolism- Glycolysis and Gluconeogenesis |
| 5226 | PGD | Carbohydrate Metabolism - Pentose Phosphate Pathway |
| 5230 | PGK1 | Carbohydrate Metabolism - Glycolysis and Gluconeogenesis |
| 5313 | PKLR | Carbohydrate Metabolism- Glycolysis/Gluconeogenesis |
| 5315 | PKM | Carbohydrate Metabolism- Glycolysis |
| 6120 | RPE | Carbon Metabolism- Pentose Phosphate Pathway |
| 6888 | TALDO1 | Carbohydrate Metabolism- Pentose Phosphate Pathway |
| 7167 | TPI1 | Carbohydrate Metabolism- Glycolysis/Gluconeogenesis |
| 8050 | PDHX | TCA Cycle and Amino Acid Metabolism |
| 8277 | TKTL1 | Carbohydrate Metabolism |
| 8402 | SLC25A11 | Carbohydrate Metabolism - Glycolysis and Gluconeogenesis |
| 8604 | SLC25A12 | Carbohydrate Metabolism - Gluconeogenesis and Amino Acids |
| 8789 | FBP2 | Carbohydrate Metabolism- Glycolysis |
| 8801 | SUCLG2 | TCA And Oxidative Phosphorylation |
| 8802 | SUCLG1 | TCA Cycle |
| 8803 | SUCLA2 | Carbohydrate Metabolism- TCA Cycle |
| 9563 | H6PDH | Carbohydrate Metabolism |
| 10873 | ME3 | TCA And Carbohydrate Metabolism |
| 22934 | RPIA | Carbohydrate Metabolism- Pentose Phosphate Path |
| 23530 | NNT | Carbohydrate Metabolism - TCA Cycle/ Redox |
| 25796 | PGLS | Carbohydrate Metabolism- Pentose Phosphate Path |
| 54704 | PPM2C | TCA Cycle and ETC |
| 55556 | ENOSF1 | Carbohydrate Metabolism |
| 57103 | C12orf5 | Carbohydrate Metabolism - Glycolysis And pentose phosphate pathway |
| 57546 | PDP2 | TCA Cycle |
| 79783 | SUGCT | Carbohydrate and TCA Metabolism |
| 79944 | L2HGDH | Carbohydrate Metabolism- TCA |
| 81889 | FAHD1 | Carbohydrate and TCA Metabolism |
| 83440 | ADPGK | Carbohydrate Metabolism- Glucose - Glycolysis and Gluconeogenesis |
| 84532 | ACSS1 | TCA Cycle/Carbon Metabolism |
| 137872 | ADHFE1 | Carbohydrate Metabolism - TCA |
| 698807 | MPC2 | TCA Cycle - Pyruvate Metabolism |
| 716864 | MPC1 | TCA Cycle |
| 97 | ACYP1 | Nucleotide Metabolism |
| 98 | ACYP2 | Nucleotide Metabolism |
| 204 | AK2 | Nucleotide Metabolism |
| 291 | SLC25A4 | Transport-Adenine Metabolism |
| 292 | SLC25A5 | Transport- Nucleotide |
| 293 | SLC25A6 | Transport - Nucleotide/Adenine Metabolism/ Apoptosis, Proteostasis |
| 1159 | CKMT1B | Amino Acid Metabolism |
| 1160 | CKMT2 | Amino Acid Metabolism |
| 1373 | CPS1 | Amino Acid Metabolism |
| 1591 | CYP24A1 | Vitamin Metabolism |
| 1594 | CYP27B1 | Vitamin Metabolism- Vitamin D |
| 1719 | DHFR | Vitamin Metabolism - Folate And 1-Carbon Metabolism/ Redox |
| 2744 | GLS | Amino Acid Metabolism- Glutamate, Neurotransmission |
| 2746 | GLUD1 | Amino Acid Metabolism / TCA Cycle |
| 4507 | MTAP | Amino Acid Metabolism |
| 4837 | NNMT | Amino Acid Metabolism- Cofactor Metabolism |
| 5009 | OTC | Amino Acid Metabolism- Creatine |
| 7298 | TYMS | Nucleotide Metabolism – Folate and 1-Carbon Metabolism/ Gene Expression |
| 10135 | NAMPT | Cofactor Metabolism - NAD/Circadian Gene Expression |
| 11194 | ABCB8 | Moonlighting - ABC/MDR Transporter |
| 23057 | NMNAT2 | Vitamin Metabolism/ NAD Metabolism |
| 23408 | SIRT5 | NAD Metabolism |
| 23409 | SIRT4 | NAD Metabolism |
| 23410 | SIRT3 | Moonlighting - NAD Metabolism - Biogenesis / Dynamics / Oxidative Phosphorylation/Protein Modification |
| 23475 | QPRT | Cofactor Metabolism - NAD |
| 27165 | GLS2 | Amino Acid Metabolism - Neurotransmitter Release |
| 27231 | NMRK2 | NAD Metabolism |
| 50808 | AK3 | Nucleoside Metabolism - Purine |
| 51074 | APIP | Amino Acid Metabolism/ Apoptosis |
| 54981 | NMRK1 | NAD Metabolism |
| 55191 | NADSYN1 | Vitamin Metabolism/NAD Metabolism |
| 56985 | ADPRM | Nucleotide Metabolism |
| 57089 | ENTPD7 | Nucleotide Metabolism |
| 60386 | SLC25A19 | Vitamins Metabolism - Transporter |
| 115286 | SLC25A26 | Amino Acid Metabolism |
| 349565 | NMNAT3 | Cofactor Metabolism - NAD |
| 548596 | CKMT1A | Amino Acid Metabolism - Urea Cycle/Redox |
| 9709 | HERPUD1 | Protein Expression and Proteostasis - UPR |
| 10245 | TIMM17B | Protein Expression and Proteostasis - Transport |
| 26517 | TIMM13 | Protein Expression and Proteostasis - Transport |
| 26519 | TIMM10 | Protein Expression and Proteostasis - Transport |
| 131474 | CHCHD4 | Protein Expression and Proteostasis - Transport |
| 10452 | TOMM40 | Protein Expression and Proteostasis - Transport/ Mitophagy |
| 56993 | TOMM22 | Protein Expression and Proteostasis - Transport/ Mitophagy |
| 9093 | DNAJA3 | Moonlighting - Protein Expression and Proteostasis - UPR/Dynamics/ Gene Expression/Apoptosis and Cell Cycle/ Stress Response |
| 1649 | DDIT3 | Protein Expression And Proteostasis - UPR/Chaperone |
| 1678 | TIMM8A | Protein Expression And Proteostasis |
| 3329 | HSPD1 | Protein Expression And Proteostasis |
| 5664 | PSEN2 | Protein Expression And Proteostasis |
| 9512 | PMPCB | Protein Expression And Proteostasis |
| 10440 | TIMM17A | Protein Expression And Proteostasis |
| 10469 | TIMM44 | Protein Expression And Proteostasis |
| 10574 | CCT7 | Protein Expression And Proteostasis |
| 26520 | TIMM9 | Protein Expression And Proteostasis |
| 26521 | TIMM8B | Protein Expression And Proteostasis |
| 29928 | TIMM22 | Protein Expression And Proteostasis |
| 1E+08 | TIMM23 | Protein Expression And Proteostasis |
| 23203 | PMPCA | Protein Expression And Proteostasis - Processing And Import |
| 3035 | HARS | Protein Expression And Proteostasis - Translation |
| 5917 | RARS | Protein Expression And Proteostasis - Translation |
| 10667 | FARS2 | Protein Expression And Proteostasis - Translation |
| 26589 | MRPL46 | Protein Expression And Proteostasis - Translation |
| 51067 | YARS2 | Protein Expression And Proteostasis - Translation |
| 51081 | MRPS7 | Protein Expression And Proteostasis - Translation |
| 51258 | MRPL51 | Protein Expression And Proteostasis - Translation |
| 51264 | MRPL27 | Protein Expression And Proteostasis - Translation |
| 51642 | MRPL48 | Protein Expression And Proteostasis - Translation |
| 51649 | MRPS23 | Protein Expression And Proteostasis - Translation |
| 55157 | DARS2 | Protein Expression And Proteostasis - Translation |
| 57129 | MRPL47 | Protein Expression And Proteostasis - Translation |
| 64432 | MRPS25 | Protein Expression And Proteostasis - Translation |
| 64960 | MRPS15 | Protein Expression And Proteostasis - Translation |
| 64969 | MRPS5 | Protein Expression And Proteostasis - Translation |
| 64975 | MRPL41 | Protein Expression And Proteostasis - Translation |
| 80324 | PUS1 | Protein Expression And Proteostasis - Translation |
| 84340 | GFM2 | Protein Expression And Proteostasis - Translation |
| 85476 | GFM1 | Protein Expression And Proteostasis - Translation |
| 90353 | CTU1 | Protein Expression And Proteostasis - Translation |
| 128308 | MRPL55 | Protein Expression And Proteostasis - Translation |
| 54938 | SARS2 | Protein Expression And Proteostasis - Translation - TRNA |
| 57038 | RARSL | Protein Expression And Proteostasis - Translation - TRNA |
| 4580 | MTX1 | Protein Expression And Proteostasis - Transport |
| 10651 | MTX2 | Protein Expression And Proteostasis - Transport |
| 54543 | TOMM7 | Protein Expression And Proteostasis - Transport |
| 25813 | SAMM50 | Protein Expression And Proteostasis - Transport And Assembly |
| 9804 | TOMM20 | Protein Expression And Proteostasis - Transport / Mitophagy |
| 3028 | HSD17B10 | Protein Expression And Proteostasis - TRNA Metabolism |
| 4141 | MARS | Protein Expression And Proteostasis - TRNA Processing, Translation/ Amino Acid Metabolism |
| 3735 | KARS | Protein Expression And Proteostasis - TRNA Processing, Translation/ Amino Acid Metabolism/ Inflammation |
| 3320 | HSP90AA1 | Protein Expression And Proteostasis / Biogenesis |
| 6182 | MRPL12 | Protein Expression And Proteostasis- Translation |
| 6183 | MRPS12 | Protein Expression And Proteostasis- Translation |
| 7284 | TUFM | Protein Expression And Proteostasis- Translation |
| 9553 | MRPL33 | Protein Expression And Proteostasis- Translation |
| 10102 | TSFM | Protein Expression And Proteostasis- Translation |
| 10884 | MRPS30 | Protein Expression And Proteostasis- Translation |
| 51021 | MRPS16 | Protein Expression And Proteostasis- Translation |
| 51023 | MRPS18C | Protein Expression And Proteostasis- Translation |
| 51253 | MRPL37 | Protein Expression And Proteostasis- Translation |
| 64928 | MRPL14 | Protein Expression And Proteostasis- Translation |
| 64951 | MRPS24 | Protein Expression And Proteostasis- Translation |
| 64963 | MRPS11 | Protein Expression And Proteostasis- Translation |
| 65003 | MRPL11 | Protein Expression And Proteostasis- Translation |
| 84545 | MRPL43 | Protein Expression And Proteostasis- Translation |
| 90480 | GADD45GIP1 | Protein Expression And Proteostasis- Translation |
| 92259 | MRPS36 | Protein Expression And Proteostasis- Translation |
| 116540 | MRPL53 | Protein Expression And Proteostasis- Translation |
| 118487 | CHCHD1 | Protein Expression And Proteostasis- Translation |
| 29074 | MRPL18 | Protein Expression And Proteostasis- Translation |
| 10128 | LRPPRC | Protein Expression And Proteostasis- Translation / OxPhos |
| 81892 | SLIRP | Protein Expression And Proteostasis- Translation / OxPhos |
| 84705 | GTPBP3 | Protein Expression And Proteostasis- Translation / Amino Acid Modification |
| 4234 | METTL1 | Protein Expression And Proteostasis- TRNA Processing, Translation |
| 92609 | TIMM50 | Protein Expression And Proteostasis/Dynamics/Apoptosis |
| 10273 | CHIP | Protein Expression And Proteostasis/Signaling |
| 3336 | HSPE1 | Protein Expression and Proteostasis - Translation |
| 3338 | DNAJC4 | Protein Expression and Proteostasis - Translation |
| 3396 | ICT1 | Protein Expression and Proteostasis - Translation |
| 4285 | MIPEP | Protein Expression and Proteostasis - Translation |
| 4287 | ATXN3 | Protein Expression and Proteostasis - Translation |
| 5018 | OXA1L | Protein Expression and Proteostasis - Translation |
| 8192 | CLPP | Protein Expression and Proteostasis - Translation |
| 8562 | DENR | Protein Expression and Proteostasis - Translation |
| 9675 | TTI1 | Protein Expression and Proteostasis - Translation |
| 9868 | TOMM70A | Protein Expression and Proteostasis - Translation |
| 10105 | PPIF | Protein Expression and Proteostasis - Translation |
| 10131 | TRAP1 | Protein Expression and Proteostasis - Translation |
| 10730 | YME1L1 | Protein Expression and Proteostasis - Translation |
| 10845 | CLPX | Protein Expression and Proteostasis - Translation |
| 10939 | AFG3L2 | Protein Expression and Proteostasis - Translation |
| 10953 | TOMM34 | Protein Expression and Proteostasis - Translation |
| 10963 | STIP | Protein Expression and Proteostasis - Translation |
| 11315 | PARK7 | Protein Expression and Proteostasis - Translation |
| 23234 | DNAJC9 | Protein Expression and Proteostasis - Translation |
| 25895 | METTL21B | Protein Expression and Proteostasis - Translation |
| 27129 | HSPB7 | Protein Expression and Proteostasis - Translation |
| 27429 | HTRA2 | Protein Expression and Proteostasis - Translation |
| 29103 | DNAJC15 | Protein Expression and Proteostasis - Translation |
| 54676 | GTPBP2 | Protein Expression and Proteostasis - Translation |
| 54927 | CHCHD3 | Protein Expression and Proteostasis - Translation |
| 55278 | QRSL1 | Protein Expression and Proteostasis - Translation |
| 55374 | TMCO6 | Protein Expression and Proteostasis - Translation |
| 55379 | LRRC59 | Protein Expression and Proteostasis - Translation |
| 57149 | LYRM1 | Protein Expression and Proteostasis - Translation |
| 78988 | MRP63 | Protein Expression and Proteostasis - Translation |
| 81570 | CLPB | Protein Expression and Proteostasis - Translation |
| 83943 | IMMP2L | Protein Expression and Proteostasis - Translation |
| 84134 | TOMM40L | Protein Expression and Proteostasis - Translation |
| 84273 | NOA1 | Protein Expression and Proteostasis - Translation |
| 135138 | PACRG | Protein Expression and Proteostasis - Translation |
| 283459 | GATC | Protein Expression and Proteostasis - Translation |
| 728489 | DNLZ | Protein Expression and Proteostasis - Translation |
| 9361 | LONP1 | Moonlighting - Protein Expression and Proteostasis - Translation/Stress Response - UPR, DNA, Redox |
| 3313 | HSPA9 | Protein Expression and Proteostasis |
| 6347 | CCL2 | Protein Expression and Proteostasis/ Stress Response - Immune Response And Chemotaxis |
| 79899 | PRR5L | Protein Expression and Proteostasis - Translation Signaling |
| 23395 | LARS2 | Protein Expression and Proteostasis - TRNA Processing, Translation |
| 23438 | HARSL | Protein Expression and Proteostasis - TRNA Processing, Translation |
| 79731 | NARS2 | Protein Expression and Proteostasis - TRNA Processing, Translation |
| 55687 | TRMU | Protein Expression and Proteostasis - TRNA Processing, Translation/Amino Acid Metabolism |
| 3337 | DNAJB1 | Protein Expression and Proteostasis- UPR - HSP Signaling/Stress Response |
| 10450 | PPIE | Protein Expression and Proteostasis- Translation, mRNA And Peptide Processing |
| 4193 | MDM2 | Moonlighting - Proteostasis/ Redox - Stress Response, Senescence, Cell Cycle, Signaling, Immune Response, P53 Signaling |
| 617 | BCS1L | Chaperone - Protein Metabolism, Proteostasis And Translation - ETC - CI, III, IV Assembly, LETM1 Complex / Dynamics |
| 581 | BAX | Stress Response- Apoptosis |
| 592 | BCL2 | Stress Response - Apoptosis And Mitophagy |
| 637 | BID | Stress Response - Apoptosis |
| 664 | BNIP3 | Stress Response - Apoptosis And Mitophagy |
| 665 | BNIP3L | Stress Response - Apoptosis And Mitophagy |
| 836 | CASP3 | Stress Response - Apoptosis |
| 2647 | BLOC1S1 | Organelle Dynamics/Organization- Membrane Trafficking |
| 4976 | OPA1 | Dynamics |
| 5071 | PARKIN | Stress Response - Apoptosis And Mitophagy |
| 6304 | SATB1 | Stress Response - Apoptosis |
| 6464 | SHC1 | Moonlighting - Stress Response/ Defense Signaling, Apoptosis, Immune /Cell Dynamics, Morphogenesis, Development, Proliferation |
| 6687 | SPG7 | Stress Response -Apoptosis And Mitophagy |
| 7157 | TP53 | Stress Response- Apoptosis, Senescence, DNA Damage Response, Cell Cycle |
| 8878 | SQSTM1 | Stress Response- Mitophagy And Apoptosis / Dynamics/ Immune Response |
| 9131 | AIFM1 | Stress Response -Apoptosis And Mitophagy |
| 9927 | MFN2 | Stress Response- Mitophagy/Turnover- Dynamics/Proteostasis, UPR |
| 10059 | DNM1L | Dynamics |
| 10989 | IMMT | Dynamics |
| 11216 | AKAP10 | Stress Response - Apoptosis/ Hemostasis |
| 22933 | SIRT2 | Stress Response -Apoptosis And Mitophagy |
| 23095 | KIF1B | Dynamics |
| 23786 | BCL2L13 | Stress Response - Apoptosis And Mitophagy |
| 28971 | AAMDC | Stress Response - Apoptosis And Mitophagy |
| 51024 | FIS1 | Dynamics |
| 51499 | TRIAP1 | Stress Response - Apoptosis And Mitophagy |
| 54332 | GDAP1 | Dynamics |
| 54471 | MIEF1 | Dynamics |
| 54708 | MARCH5 | Dynamics |
| 55210 | ATAD3A | Dynamics |
| 55288 | RHOT1 | Dynamics |
| 55486 | PARL | Stress Response - Apoptosis And Mitophagy |
| 55669 | MFN1 | Dynamics - Fusion, Mitophagy, Turnover |
| 55735 | DNAJC11 | Dynamics |
| 56616 | DIABLO | Stress Response- Apoptosis |
| 56947 | MFF | Dynamics |
| 57761 | TRIB3 | Stress Response - Apoptosis |
| 64798 | DEPTOR | Stress Response -Apoptosis And Mitophagy |
| 79594 | MUL1 | Dynamics |
| 83858 | ATAD3B | Dynamics |
| 84303 | CHCHD6 | Dynamics |
| 84902 | CEP89 | Dynamics |
| 89941 | RHOT2 | Dynamics And Organization |
| 99501 | PINK1 | Stress Response - Mitophagy/Macroautophagy |
| 115209 | OMA1 | Dynamics |
| 125170 | MIEF2 | Dynamics |
| 150962 | PUS10 | Stress Response - Apoptosis And Mitophagy |
| 192111 | PGAM5 | Stress Response - Apoptosis And Mitophagy |
| 219293 | ATAD3C | Dynamics |
| 253260 | RICTOR | Moonlighting - Cell Response/ Signaling/ Nutrient Response/ Proteostasis/Inflammatory And Immune System |
| 440574 | MINOS1 | Dynamics |
| 100463289 | MTRNR2L5 | Stress Response - Apoptosis And Mitophagy |
| 3954 | LETM1 | Calcium Signaling And Homeostasis -Calcium Transporter/Dynamics |
| 10367 | CBARA1 | Calcium Signaling And Homeostasis - Transporter |
| 90550 | CCDC109A | Calcium Signaling And Homeostasis - Transporter |
| 91689 | SMDT1 | Calcium Signaling And Homeostasis - Transporter |
| 101128054 | MICU2 | Calcium Signaling And Homeostasis - Transporter |
| 811 | CALR | Calcium Signaling - Signal Transduction/Immune Response |
| 3708 | ITPR1 | Calcium Signaling - Signal Transduction/Immune Response/ Stress Response - Apoptosis |
| 488 | ATP2A2 | Calcium Signaling And Homeostasis - Transporter |
| 489 | ATP2A3 | Calcium Signaling And Homeostasis - Transporter |
| 5498 | PPOX | Heme Metabolism/Redox |
| 51312 | SLC25A37 | Iron-Sulfur Cluster Biogenesis |
| 9054 | NFS1 | Iron-Sulfur Cluster Biogenesis/ Amino Acid Metabolism |
| 7416 | VDAC1 | Moonlighting - Calcium Transport/ Mitophagy And Apoptosis /Redox |
| 1728 | NQO1 | Redox |
| 27035 | NOX1 | Redox |
| 84816 | RTN4IP1 | Redox |
| 10811 | NOXA1 | Redox |
| 124056 | NOXO1 | Redox |
| 25974 | MMACHC | Redox /Vitamin Metabolism And Cofactors |
| 2730 | GCLM | Redox - Glutathione Antioxidant Synthesis / Amino Acid Metabolism/ Apoptosis |
| 3091 | HIF1A | Redox - Hypoxia Stress Response |
| 112399 | EGLN3 | Moonlighting - Redox - Hypoxia Stress Response / Proteostasis - Protein Degradation |
| 48 | ACO1 | Redox - Iron Metabolism |
| 3658 | IREB2 | Redox - Iron Metabolism |
| 55847 | CISD1 | Redox - Iron Metabolism |
| 2395 | FXN | Redox - Iron-Sulfur Cluster/ Proteostasis |
| 23479 | ISCU | Redox - Iron-Sulfur Cluster/ Proteostasis |
| 57128 | LYRM4 | Redox - Iron-Sulfur Cluster/ Proteostasis |
| 27247 | NFU1 | Redox - Iron-Sulfur Metabolism And Heme |
| 81689 | HBLD2 | Redox - Iron-Sulfur Metabolism And Heme |
| 122961 | HBLD1 | Redox - Iron-Sulfur Metabolism And Heme |
| 200205 | IBA57 | Redox - Iron-Sulfur Metabolism And Heme |
| 493856 | CISD2 | Redox - Iron-Sulfur Metabolism And Heme |
| 150274 | HSCB | Redox - Iron-Sulfur Cluster/ Proteostasis |
| 4780 | NFE2L2 | Redox - Stress Response |
| 140823 | ROMO1 | Redox - Stress Response |
| 5250 | SLC25A3 | Redox - Transporter |
| 25932 | CLIC4 | Redox - Transporter |
| 4842 | NOS1 | Redox - Ion Homeostasis |
| 240 | ALOX5 | Redox - Stress Response - Lipoxygenase Metabolism / Lipid Metabolism |
| 7001 | PRDX2 | Redox Stress Response- Antioxidant, Inflammatory & Immune Response |
| 1536 | CYBB | Redox Stress Response- Defense Response, Immune & Inflammatory Response |
| 9588 | PRDX6 | Redox Stress Response- Detoxification |
| 25828 | TXN2 | Redox Stress Response- Detoxification |
| 4968 | OGG1 | Redox Stress Response- DNA Damage Response, DNA Repair |
| 4843 | NOS2 | Redox- Defense Response, Immune & Inflammatory Response, Hemostasis |
| 25824 | PRDX5 | Redox - Oxidative Stress Response |
| 3162 | HMOX1 | Redox - Porphyrin And Heme Metabolism, Hypoxia Response |
| 4846 | NOS3 | Redox - Production Of ROS/RNS, Signaling, Vasodilation, Hemostasis, Morphogenesis, Ion Transport |
| 5052 | PRDX1 | Redox - Stress Response, Detoxification, Antioxidant |
| 6648 | SOD2 | Redox - Stress Response, Detoxification, Antioxidant |
| 6649 | SOD3 | Redox - Stress Response, Detoxification, Antioxidant |
| 10935 | PRDX3 | Redox - Stress Response, Detoxification, Antioxidant |
| 7295 | TXN | Redox - Stress Response, Detoxification, Gene Expression, Inflammation, Signaling, Metabolism |
| 6647 | SOD1 | Redox - Stress Response, Hemostasis |
| 1535 | CYBA | Redox - Stress Response, Inflammatory & Immune Response |
| 2671 | GFER | Redox/Proteostasis |
| 7350 | UCP1 | Redox/ETC/Transport |
| 7351 | UCP2 | Redox/ETC/Transport |
| 7352 | UCP3 | Redox/ETC/Transport |
| 4482 | MSRA | Redox/Proteostasis |
| 22921 | MSRB2 | Redox/Proteostasis |
| 2876 | GPX1 | Redox - Stress Response/Lipid Metabolism |
| 2879 | GPX4 | Redox - Stress Response/Lipid Metabolism |
| 8165 | AKAP1 | Signaling - PKA, Hemostasis |
| 4358 | MPV17 | Stress Response - DNA Damage And Repair/Redox |
| 4595 | MUTYH | Stress Response - DNA Damage And Repair/Redox |
| 8846 | ALKBH1 | Stress Response - DNA Damage And Repair/Redox |
| 121642 | ALKBH2 | Stress Response - DNA Damage And Repair/Redox |
| 221120 | ALKBH3 | Stress Response - DNA Damage And Repair/Redox |
| 10280 | SIGMAR1 | Moonlighting - Stress Response - Redox, Apoptosis/DNA And Protein Metabolism/Proteostasis And Translation |
| 4521 | NUDT1 | Stress Response - Redox - DNA Damage And Repair/Nucleotide Metabolism |
| 7444 | VRK2 | Stress Response/Redox- Immune And Inflammatory Response, Apoptosis, Hypoxia/ Cell Cycle |
| 51076 | CUTC | Transport- Ion, Copper |
| 23456 | ABCB10 | Transporter - ABC |
| 10058 | ABCB6 | Transporter - Heme And Porphyrin |
| 142 | PARP1 | Gene Expression - Stress Response - DNA Repair/ Apoptosis/ Oxidative Phosphorylation |
| 1026 | CDKN1A | Gene Expression - Stress Response- DNA Damage Response, Cell Cycle Arrest, Senescence/ Apoptosis |
| 1051 | CEBPB | Moonlighting- Stress Response/Gene Expression/ Inflammatory Response And Immune Response- Senescence And SASP |
| 1654 | DDX3X | Stress Response - DNA Damage And Repair / Apoptosis |
| 2189 | FANCG | Stress Response/DNA Metabolism - DNA Repair, Gene Expression |
| 2475 | MTOR | Moonlighting - Stress Response/Nutrient Signaling/ Metabolic Integration/ Autophagy/Gene Expression - DNA Damage Response/ Immune Response |
| 3021 | H3F3B | Stress Response - DNA Damage And Repair |
| 3980 | LIG3 | Stress Response- DNA Repair |
| 7374 | UNG | Stress Response- Gene Expression - DNA Repair |
| 10038 | PARP2 | Stress Response- Gene Expression - DNA Repair |
| 27301 | APEX2 | Stress Response - DNA Damage And Repair |
| 54541 | DDIT4 | Stress Response - Signaling And Gene Expression |
| 80119 | PIF1 | Stress Response - DNA Damage And Repair |
| 83667 | SESN2 | Moonlighting- Stress Response/Gene Expression/ Metabolic Regulation/ Amino Acid Sensor/Signaling/ |
| 201973 | PRIMPOL | Stress Response - DNA Damage And Repair |
| 468 | ATF4 | Moonlighting- Gene Expression / Proteostasis And Protein Metabolism / Apoptosis/ Carbohydrate And Amino Acid Metabolism |
| 891 | CCNB1 | Gene Expression- Damage Response, Cell Cycle |
| 983 | CDK1 | Gene Expression - Cell Cycle/ DNA Repair/ Biogenesis |
| 995 | CDC25C | Gene Expression - Cell Cycle/DNA Damage Response/ Stress Response |
| 1022 | CDK7 | Gene Expression - Transcription Regulation, mRNA Regulation, DNA Repair, Cell Cycle |
| 1050 | CEBPA | Moonlighting - Gene Expression/Differentiation And Development/Fat And Adipose Tissue/Metabolic Integration/ Proteostasis |
| 1053 | CEBPE | Gene Expression - DNA Metabolism And Maintenance |
| 1385 | CREB1 | Gene Expression/Signal Transduction |
| 1386 | ATF2 | Moonlighting - Gene Expression - Biogenesis / Stress Response Signaling-Immune Response, Apoptosis |
| 1716 | DGUOK | Gene Expression- Nucleotide Metabolism |
| 1763 | DNA2 | Gene Expression- DNA/Nucleotide Metabolism, Repair, Cell Cycle |
| 1854 | DUT | Gene Expression- DNA Metabolism - Pyrimidine Metabolism |
| 1890 | TYMP | Gene Expression- DNA Metabolism - Pyrimidine Metabolism And Genome Metabolism |
| 1982 | EIF4G2 | Gene Expression- Translation Regulation, Immune Response, Cell Growth And Cycle |
| 2002 | ELK1 | Gene Expression - Transcription/ Immune System/ Signaling |
| 2021 | ENDOG | Gene Expression- DNA Metabolism And Maintenance |
| 2101 | ESRRA | Gene Expression- Biogenesis, Cell Proliferation |
| 2103 | ESRRB | Gene Expression- Stem Cell Regulation, Cell Cycle, Metabolism |
| 2104 | ESRRG | Gene Expression - Transcription |
| 2305 | FOXM1 | Gene Expression- Nucleotide Metabolism, Repair, Cell Cycle |
| 2551 | GABPA | Gene Expression - Biogenesis / Dynamics |
| 4899 | NRF1 | Gene Expression-DNA Metabolism And Maintenance/ Biogenesis/Lipid Metabolism Regulation PPARa |
| 5000 | ORC4 | Gene Expression - Cell Cycle And DNA Replication |
| 5245 | PHB | Moonlighting- Gene Expression/ Stress Response - Inflammatory And Immune System/Signaling - Calcium / Dynamics - Mitochondrial Organization |
| 5428 | POLG | Gene Expression, DNA Metabolism And Maintenance |
| 5442 | POLRMT | Gene Expression - Biogenesis |
| 5468 | PPARG | Gene Expression - Metabolic Integration/Biogenesis /Regulation Of Lipid Metabolism - |
| 5889 | RAD51C | DNA Metabolism/Stress Response- DNA Repair, Gene Expression, Cell Cycle, Meiosis |
| 6742 | SSBP1 | Gene Expression - Biogenesis / Dynamics |
| 6832 | SUPV3L1 | Gene Expression, DNA Metabolism And Maintenance |
| 7019 | TFAM | Gene Expression - Biogenesis / Dynamics |
| 7084 | TK2 | Gene Expression- DNA/Nucleotide Metabolism, Cell Cycle |
| 8467 | SMARCA5 | Gene Expression- DNA Repair/ Cell Cycle/ Stress Response |
| 10891 | PPARGC1A | Moonlighting- Regulation Of Turnover - Biogenesis And Mitophagy/Dynamics/ Energy Metabolism - Lipids And Response To Nutrient |
| 11232 | POLG2 | Gene Expression - Biogenesis/ DNA Repair/ Dynamics |
| 11331 | PHB2 | Gene Expression, DNA Metabolism And Maintenance |
| 23314 | SATB2 | Gene Expression, DNA Metabolism And Maintenance |
| 29960 | FTSJ2 | Gene Expression, DNA Metabolism And Maintenance |
| 50484 | RRM2B | Gene Expression- Nucleotide Metabolism, DNA Replication, DNA Repair, Redox, Apoptosis |
| 51106 | TFB1M | Gene Expression - Biogenesis/ DNA Repair/ Dynamics |
| 54617 | INO80 | Gene Expression, DNA Metabolism And Maintenance, DNA Repair |
| 54790 | TET2 | Gene Expression- Epigenetic Regulation, Transcription Regulation |
| 55149 | PAPD1 | Gene Expression, DNA Metabolism And Maintenance |
| 56652 | C10orf2 | Gene Expression, DNA Metabolism And Maintenance |
| 64216 | TFB2M | Gene Expression - Transcription - Biogenesis And Maintenance |
| 84808 | PERM1 | Gene Expression, DNA Metabolism And Maintenance |
| 87178 | PNPT1 | Gene Expression, DNA Metabolism And Maintenance |
| 92667 | C20orf72 | Gene Expression, DNA Metabolism And Maintenance |
| 155061 | ZNF746 | Gene Expression |
| 10328 | EMC8 | Unknown |
| 57226 | LYRM2 | Unknown |
| 728568 | C12ORF73 | Unknown |

Table S3. The longevity-associated gene set and the evolutionary conservation of their longevity-associations (SKAT p≤0.050).

| Number | Gene Symbol | P-value | Yeast | Worm | Fly | Mouse |
| --- | --- | --- | --- | --- | --- | --- |
| 1 | PARP2* | 1.54E-03 |  |  |  |  |
| 2 | ENO1^#^ | 1.60E-03 |  |  |  |  |
| 3 | TP53* | 2.34E-03 |  |  |  | + |
| 4 | SMARCA5^#^ | 2.34E-03 | - |  |  |  |
| 5 | MRPS25^#^ | 2.44E-03 |  |  |  |  |
| 6 | LETM1 | 2.59E-03 |  | - |  |  |
| 7 | ACADM | 2.69E-03 |  |  |  |  |
| 8 | APIP* | 3.10E-03 |  |  |  |  |
| 9 | ME2 | 3.13E-03 |  |  | + |  |
| 10 | GFM1 | 3.21E-03 |  |  |  |  |
| 11 | MTAP | 4.03E-03 |  |  |  |  |
| 12 | SLC27A5* | 4.56E-03 |  |  |  |  |
| 13 | HMGCL | 5.04E-03 |  |  |  |  |
| 14 | HMGCS1* | 5.08E-03 |  |  |  |  |
| 15 | NDUFB10* | 5.35E-03 |  |  | - |  |
| 16 | GPD2 | 6.04E-03 | + |  |  |  |
| 17 | ACACA | 6.77E-03 |  |  |  |  |
| 18 | TALDO1* | 6.87E-03 | - | - |  |  |
| 19 | ALDH3B1 | 8.61E-03 |  |  |  |  |
| 20 | NFU1^#^ | 9.21E-03 | + |  |  |  |
| 21 | MFF | 1.04E-02 |  |  |  |  |
| 22 | MRPL51^#^ | 1.23E-02 |  |  |  |  |
| 23 | PLA2G15* | 1.36E-02 |  |  |  |  |
| 24 | CYCS | 1.45E-02 |  |  |  |  |
| 25 | CKMT1A^#^ | 1.56E-02 |  | - |  |  |
| 26 | NDUFA3 | 1.58E-02 |  |  |  |  |
| 27 | ACSL4^#^ | 1.69E-02 |  | - |  |  |
| 28 | CKMT2^#^ | 1.70E-02 |  |  |  |  |
| 29 | BNIP3^#^ | 1.80E-02 |  |  |  |  |
| 30 | DLST | 1.85E-02 |  |  |  |  |
| 31 | PIP4K2B | 1.99E-02 |  |  |  |  |
| 32 | ATP5S | 2.05E-02 |  |  |  |  |
| 33 | MFN2^#^ | 2.08E-02 |  |  |  |  |
| 34 | GABPA^#^ | 2.09E-02 |  |  |  |  |
| 35 | COX18^#^ | 2.12E-02 |  |  |  |  |
| 36 | SPG7^#^ | 2.14E-02 | - |  |  |  |
| 37 | CHCHD6 | 2.19E-02 |  |  |  |  |
| 38 | CYC1* | 2.23E-02 | - |  |  |  |
| 39 | PPARG* | 2.27E-02 |  |  |  | + |
| 40 | BDH1^#^ | 2.30E-02 |  |  |  |  |
| 41 | TOMM40L | 2.37E-02 |  |  |  |  |
| 42 | ACAT2^#^ | 2.39E-02 |  |  |  |  |
| 43 | FOXM1^#^ | 2.42E-02 | + |  |  | + |
| 44 | PKLR^#^ | 2.47E-02 |  |  |  |  |
| 45 | RARS^#^ | 2.48E-02 |  | - |  |  |
| 46 | SLC27A2^#^ | 2.50E-02 | + |  |  |  |
| 47 | KDSR^#^ | 2.50E-02 |  |  |  |  |
| 48 | ACSM1 | 2.59E-02 |  |  |  |  |
| 49 | H3F3B | 2.60E-02 |  | + |  |  |
| 50 | COX5B^#^ | 2.70E-02 |  | - |  |  |
| 51 | ATP5E | 2.72E-02 |  |  |  |  |
| 52 | COQ6 | 2.74E-02 |  |  |  |  |
| 53 | CYP11B2 | 2.88E-02 |  |  |  |  |
| 54 | EIF4G2 | 2.90E-02 | - | - |  |  |
| 55 | PDK4 | 2.91E-02 |  |  |  |  |
| 56 | GATC^#^ | 2.98E-02 |  |  |  |  |
| 57 | FTSJ2 | 2.99E-02 |  |  |  |  |
| 58 | PGK1 | 3.19E-02 |  |  |  |  |
| 59 | HMGCS2 | 3.23E-02 |  |  |  |  |
| 60 | ECHDC3 | 3.25E-02 |  |  |  |  |
| 61 | MRPS36 | 3.33E-02 |  |  |  |  |
| 62 | NDUFC1^#^ | 3.56E-02 |  |  |  |  |
| 63 | AACS | 3.96E-02 | + |  |  |  |
| 64 | UNG^#^ | 3.97E-02 |  |  |  |  |
| 65 | GCK | 4.12E-02 |  |  | - |  |
| 66 | MSRA | 4.22E-02 | + |  | + | + |
| 67 | OGDH | 4.22E-02 | + | - |  |  |
| 68 | PDHB | 4.26E-02 |  |  |  |  |
| 69 | FIS1^#^ | 4.26E-02 | - |  |  |  |
| 70 | DDX3X | 4.48E-02 |  |  |  |  |
| 71 | SLC25A4 | 4.53E-02 | +/- | - |  | + |
| 72 | ATAD3C | 4.56E-02 |  | - |  |  |
| 73 | HSPB7 | 4.82E-02 |  |  |  |  |
| 74 | ACER2 | 4.87E-02 |  |  |  |  |
| 75 | TIMM9 | 4.95E-02 |  |  |  |  |
| 76 | SDHAF2 | 4.99E-02 | - |  |  |  |

^#^Genes significant in 2 of the 3 SKATs; *Genes significant in all three SKATs. Lowest p-value test result reported for genes significant in multiple tests. Model organism data indicates directionality of lifespan phenotype resulting from gene manipulation: (+) indicates a pro-longevity phenotype in the indicated model organism; (-) indicates an anti-longevity phenotype in the indicated model organism. SKAT results shown separately see **Table** **S4.**

Table S4. Longevity-associated mitonuclear genes identified by different types of SKAT (p≤0.050).

| **SKAT - O** | | **SKAT** | | **SKAT-C** | |
| --- | --- | --- | --- | --- | --- |
| **Gene Symbol** | **P-value** | **Gene Symbol** | **P-value** | **Gene Symbol** | **P-value** |
| TP53* | 2.34E-03 | ENO1^#^ | 1.60E-03 | PARP2* | 1.54E-03 |
| MRPS25^#^ | 2.44E-03 | SMARCA5^#^ | 2.34E-03 | ME2 | 3.13E-03 |
| LETM1 | 2.59E-03 | SLC27A5* | 4.56E-03 | GFM1 | 3.21E-03 |
| ACADM | 2.69E-03 | TP53* | 5.20E-03 | MTAP | 4.03E-03 |
| APIP* | 3.10E-03 | NDUFB10* | 5.35E-03 | TP53* | 5.04E-03 |
| SMARCA5^#^ | 4.48E-03 | TALDO1* | 6.87E-03 | HMGCL | 5.04E-03 |
| ENO1^#^ | 4.70E-03 | NFU1^#^ | 9.21E-03 | HMGCS1* | 5.08E-03 |
| NDUFB10* | 5.88E-03 | MRPL51^#^ | 1.23E-02 | NDUFB10* | 5.40E-03 |
| GPD2 | 6.04E-03 | PLA2G15* | 1.36E-02 | ALDH3B1 | 8.61E-03 |
| ACACA | 6.77E-03 | PARP2* | 1.38E-02 | TALDO1* | 9.27E-03 |
| PARP2* | 6.91E-03 | CKMT1A^#^ | 1.56E-02 | NDUFA3 | 1.58E-02 |
| SLC27A5* | 7.04E-03 | ACSL4^#^ | 1.69E-02 | PIP4K2B | 1.99E-02 |
| MFF | 1.04E-02 | CKMT2^#^ | 1.70E-02 | ATP5S | 2.05E-02 |
| TALDO1* | 1.38E-02 | BNIP3^#^ | 1.80E-02 | CYC1* | 2.33E-02 |
| NFU1^#^ | 1.39E-02 | MRPS25^#^ | 1.88E-02 | TOMM40L | 2.37E-02 |
| CYCS | 1.45E-02 | HMGCS1* | 1.95E-02 | PDK4 | 2.91E-02 |
| DLST | 1.85E-02 | MFN2^#^ | 2.08E-02 | SLC27A5* | 2.93E-02 |
| CHCHD6 | 2.19E-02 | GABPA^#^ | 2.09E-02 | PPARG* | 3.11E-02 |
| MRPL51^#^ | 2.31E-02 | COX18^#^ | 2.12E-02 | ECHDC3 | 3.25E-02 |
| CYC1* | 2.36E-02 | SPG7^#^ | 2.14E-02 | APIP* | 3.38E-02 |
| RARS^#^ | 2.48E-02 | CYC1* | 2.23E-02 | NDUFC1^#^ | 3.69E-02 |
| PLA2G15* | 2.50E-02 | PPARG* | 2.27E-02 | SLC27A2^#^ | 3.79E-02 |
| CKMT1A^#^ | 2.57E-02 | BDH1^#^ | 2.30E-02 | AACS | 3.96E-02 |
| ACSM1 | 2.59E-02 | ACAT2^#^ | 2.39E-02 | MSRA | 4.22E-02 |
| H3F3B | 2.60E-02 | FOXM1^#^ | 2.42E-02 | FIS1^#^ | 4.26E-02 |
| ATP5E | 2.72E-02 | PKLR^#^ | 2.47E-02 | PLA2G15* | 4.55E-02 |
| CYP11B2 | 2.88E-02 | SLC27A2^#^ | 2.50E-02 | HSPB7 | 4.82E-02 |
| GATC^#^ | 2.98E-02 | KDSR^#^ | 2.50E-02 | TIMM9 | 4.95E-02 |
| ACSL4^#^ | 3.12E-02 | APIP* | 2.57E-02 | SDHAF2 | 4.99E-02 |
| CKMT2^#^ | 3.17E-02 | COX5B^#^ | 2.70E-02 |  |  |
| PGK1 | 3.19E-02 | COQ6 | 2.74E-02 |  |  |
| ACAT2^#^ | 3.24E-02 | RARS^#^ | 2.90E-02 |  |  |
| BNIP3^#^ | 3.47E-02 | EIF4G2 | 2.90E-02 |  |  |
| FOXM1^#^ | 3.51E-02 | FTSJ2 | 2.99E-02 |  |  |
| MFN2^#^ | 3.53E-02 | HMGCS2 | 3.23E-02 |  |  |
| HMGCS1* | 3.58E-02 | MRPS36 | 3.33E-02 |  |  |
| COX18^#^ | 3.69E-02 | NDUFC1^#^ | 3.56E-02 |  |  |
| SPG7^#^ | 3.78E-02 | UNG^#^ | 3.97E-02 |  |  |
| PPARG* | 4.14E-02 | GCK | 4.12E-02 |  |  |
| OGDH | 4.22E-02 | PDHB | 4.26E-02 |  |  |
| GABPA^#^ | 4.35E-02 | SLC25A4 | 4.53E-02 |  |  |
| COX5B^#^ | 4.48E-02 | FIS1^#^ | 4.60E-02 |  |  |
| DDX3X | 4.48E-02 | GATC^#^ | 4.69E-02 |  |  |
| PKLR^#^ | 4.49E-02 |  |  |  |  |
| BDH1^#^ | 4.52E-02 |  |  |  |  |
| ATAD3C | 4.56E-02 |  |  |  |  |
| UNG^#^ | 4.78E-02 |  |  |  |  |
| ACER2 | 4.87E-02 |  |  |  |  |
| KDSR^#^ | 4.89E-02 |  |  |  |  |

Results listed in order of most to least significant’; ^#^Genes significant in 2 of the 3 SKATs; *Genes significant in all three SKATs

Table S5. Enriched GO terms in longevity-associated genes (SKAT≤0.050).

| GO terms enriched against the human whole genome (~21,000 genes) | Gene# (REF) | Gene# (QUERY) | Fold Enrichment | P-value |
| --- | --- | --- | --- | --- |
| ketone body biosynthetic process | 9 | 5 | > 100 | 1.55E-06 |
| ketone body metabolic process | 12 | 5 | > 100 | 4.69E-06 |
| cellular ketone body metabolic process | 11 | 5 | > 100 | 4.69E-06 |
| mitochondrial ATP synthesis coupled proton transport | 21 | 4 | 51.27 | 1.81E-02 |
| acetyl-CoA metabolic | 29 | 5 | 46.41 | 1.37E-03 |
| NADH metabolic process | 36 | 6 | 44.86 | 8.72E-05 |
| NAD regeneration | 25 | 4 | 43.07 | 3.36E-02 |
| GO terms enriched against the whole mitochondrial proteome (~2,220 genes) | Gene# (REF) | Gene# (QUERY) | Fold Enrichment | P-value |
| ketone body biosynthetic process | 5 | 5 | 28.04 | 2.33E-02 |
| cellular ketone body metabolic process | 6 | 5 | 23.37 | 4.15E-02 |
| ketone body metabolic process | 6 | 5 | 23.37 | 4.15E-02 |
| regulation of cell growth | 37 | 9 | 6.82 | 4.16E-02 |
| GO terms enriched against the mitonuclear candidate list (~660 genes) | Gene# (REF) | Gene# (QUERY) | Fold Enrichment | P-value* |
| ketone body biosynthetic process | 6 | 5 | 7.24 | n.s. |
| ketone body metabolic process | 6 | 5 | 7.24 | n.s. |
| cellular ketone body metabolic process | 6 | 5 | 3.2 | n.s. |

Terms listed represent functional clusters with positive fold enrichment reported by hierarchical clustering analysis for complete biological process GO terms with the online PANTHER tool. P-values reported are Bonferroni corrected for multiple testing. *P-values reported are not-significant (n.s.); Reference (REF) background gene list; Query (QUERY) gene list = SKAT longevity- associated genes (p≤0.050).

Table S6. 112 prioritized LAVs after SNP-based analysis

| Gene | Variant rsID^+^ | Type | Transcript ID^#^ | Nucleotide Change^#^ | Amino Acid Change^#^ | MAF Case^%^ | MAF Control^%^ | Direction^&^ | P |
| --- | --- | --- | --- | --- | --- | --- | --- | --- | --- |
| NQO1 | rs689454 | upstream gene | ENST00000320623 |  |  | 0.1631 | 0.0847 | Enriched | 1.07E-06 |
| NOS1 | rs9658570 | 3 prime UTR | ENST00000317775.6 | c.*2241T>G |  | 0.0837 | 0.0385 | Enriched | 1.32E-05 |
| SUCLG2 | rs116261805 | upstream gene | ENST00000493112 |  |  | 0.0499 | 0.0166 | Enriched | 3.11E-05 |
| MFF | . | intron | ENST00000353339.3 | c.752+85G>T |  | 0.0121 | 0.0009 | Enriched | 0.0010 |
| OXA1L | rs8572 | missense | ENST00000285848.5 | c.311C>T | p.Val104Ala | 0.1465 | 0.2013 | Depleted | 0.0029 |
| CYC1 | rs180771272 | upstream gene | ENST00000318911 |  |  | 0.0082 | 0.0000 | Enriched | 0.0029 |
| AACS | rs7138557 | intron | ENST00000316519.6 | c.767+79T>C |  | 0.4466 | 0.3829 | Enriched | 0.0031 |
| AACS | rs56394386 | intron | ENST00000316519.6 | c.767+1093A>G |  | 0.4385 | 0.3767 | Enriched | 0.0041 |
| LRPPRC | rs146630100 | missense | ENST00000260665.7 | c.4056T>A | p.Asp1352Glu | 0.0071 | 0.0000 | Enriched | 0.0046 |
| COX5B | . | upstream gene | ENST00000258424 |  |  | 0.0071 | 0.0000 | Enriched | 0.0046 |
| TOMM40 | rs11556505 | synonymous | ENST00000252487.5 | c.393C>T | p.Phe131= | 0.0529 | 0.0855 | Depleted | 0.0058 |
| HK2 | rs10194657 | synonymous | ENST00000290573.2 | c.2298A>G | p.Leu766= | 0.4546 | 0.3960 | Enriched | 0.0065 |
| ATP5S | rs12433794 | missense | ENST00000358473.1 | c.53A>G | p.Glu18Gly | 0.5121 | 0.4537 | Enriched | 0.0071 |
| SLC27A5 | . | upstream gene | ENST00000263093 |  |  | 0.0111 | 0.0017 | Enriched | 0.0073 |
| MFF | . | upstream gene | ENST00000353339 |  |  | 0.0091 | 0.0009 | Enriched | 0.0077 |
| NDUFC1 | . | 5 prime UTR | ENST00000394223.1 | c.-278C>T |  | 0.0141 | 0.0035 | Enriched | 0.0084 |
| TOMM20 | . | missense | ENST00000366607.4 | c.418G>A | p.Ala140Thr | 0.0000 | 0.0070 | Depleted | 0.0088 |
| NDUFS1 | . | missense | ENST00000233190.6 | c.848A>G | p.Glu283Gly | 0.0000 | 0.0070 | Depleted | 0.0088 |
| CYCS | . | downstream gene | ENST00000305786 |  |  | 0.0071 | 0.0000 | Enriched | 0.0092 |
| DLST | . | upstream gene | ENST00000334220 |  |  | 0.0356 | 0.0166 | Enriched | 0.0095 |
| PCK1 | rs2070756 | synonymous | ENST00000319441.4 | c.1140T>C | p.Gly380= | 0.3990 | 0.3418 | Enriched | 0.0095 |
| PDHB | . | intron | ENST00000383714.4 | c.536-29A>T |  | 0.0060 | 0.0000 | Enriched | 0.0100 |
| STIP1 | rs151183233 | synonymous | ENST00000305218.4 | c.81C>T | p.Tyr27= | 0.0060 | 0.0000 | Enriched | 0.0100 |
| YARS2 | rs35339227 | synonymous | ENST00000324868.8 | c.1026G>A | p.Arg342= | 0.0060 | 0.0000 | Enriched | 0.0100 |
| ME2 | rs674044 | upstream gene | ENST00000321341 |  |  | 0.3357 | 0.2850 | Enriched | 0.0128 |
| NOX1 | rs142303829 | missense | ENST00000372966.3 | c.721C>T | p.Arg241Cys | 0.0786 | 0.0516 | Enriched | 0.0130 |
| RARS | rs78655764 | upstream gene | ENST00000231572 |  |  | 0.0383 | 0.0201 | Enriched | 0.0131 |
| SUCLA2 | rs771276854 | missense | ENST00000378654.3 | c.851G>A | p.Arg284His | 0.0010 | 0.0087 | Depleted | 0.0137 |
| ABCB8 | rs139216778 | missense | ENST00000356058.4 | c.415C>T | p.Pro139Ser | 0.0000 | 0.0100 | Depleted | 0.0139 |
| Gene | Variant rsID^+^ | Type | Transcript ID^#^ | Nucleotide Change^#^ | Amino Acid Change^#^ | MAF Case^%^ | MAF Control^%^ | Direction^&^ | P |
| COX5B | rs17022045 | upstream gene | ENST00000258424 |  |  | 0.1663 | 0.1285 | Enriched | 0.0141 |
| ACAT2 | rs6937779 | intron | ENST00000367048.4 | c.190+71A>T |  | 0.0725 | 0.0457 | Enriched | 0.0154 |
| HMGCS2 | . | intron | ENST00000369406.3 | c.105-1136A>G |  | 0.0161 | 0.0052 | Enriched | 0.0167 |
| ACADM | . | missense | ENST00000541113.1 | c.307G>A | p.Asp103Asn | 0.0000 | 0.0061 | Depleted | 0.0171 |
| AACS | rs374884365 | missense | ENST00000316519.6 | c.619G>A | p.Val207Met | 0.0000 | 0.0061 | Depleted | 0.0171 |
| UQCC | . | missense | ENST00000374385.5 | c.541G>A | p.Glu181Lys | 0.0000 | 0.0061 | Depleted | 0.0171 |
| PFKM | rs2228500 | missense | ENST00000340802.6 | c.512G>A | p.Arg171Gln | 0.2319 | 0.2762 | Depleted | 0.0195 |
| MFF | rs6436682 | upstream gene | ENST00000353339 |  |  | 0.0383 | 0.0210 | Enriched | 0.0198 |
| GLS2 | rs2657879 | missense | ENST00000311966.4 | c.1742T>C | p.Leu581Pro | 0.1371 | 0.1740 | Depleted | 0.0200 |
| KIF1B | rs2297881 | missense | ENST00000377086.1 | c.3398A>G | p.Tyr1133Cys | 0.0292 | 0.0490 | Depleted | 0.0200 |
| COX15 | rs2231687 | missense | ENST00000370483.5 | c.1120T>C | p.Leu374Phe | 0.2117 | 0.1713 | Enriched | 0.0202 |
| APIP | rs113604896 | intron | ENST00000395787.3 | c.57+487dupT |  | 0.0837 | 0.0577 | Enriched | 0.0213 |
| ACAT2 | rs756289468 | 5 prime UTR | ENST00000367048.4 | c.-1016G>A |  | 0.0050 | 0.0000 | Enriched | 0.0215 |
| ITPR1 | . | missense | ENST00000443694.2 | c.7295G>T | p.Arg2432Leu | 0.0050 | 0.0000 | Enriched | 0.0215 |
| PDP1 | . | missense | ENST00000396200.3 | c.1625A>T | p.Asp542Val | 0.0050 | 0.0000 | Enriched | 0.0215 |
| GFM1 | rs114379664 | upstream gene | ENST00000486715 |  |  | 0.0050 | 0.0000 | Enriched | 0.0215 |
| HMGCS1 | . | upstream gene | ENST00000325110 |  |  | 0.0050 | 0.0000 | Enriched | 0.0215 |
| ACSM1 | . | intron | ENST00000520010.1 | c.-51-2041G>A |  | 0.0050 | 0.0000 | Enriched | 0.0215 |
| LRPPRC | rs149693840 | missense | ENST00000260665.7 | c.4132A>G | p.Ser1378Gly | 0.0050 | 0.0000 | Enriched | 0.0215 |
| ALDOA | . | stop gained | ENST00000569798.1 | c.1048G>T | p.Glu350Ter | 0.0068 | 0.0000 | Enriched | 0.0219 |
| ACAT2 | rs55741530 | 5 prime UTR | ENST00000367048.4 | c.-385A>T |  | 0.0477 | 0.0267 | Enriched | 0.0220 |
| APIP | rs3763932 | intron | ENST00000395787.3 | c.57+412G>A |  | 0.1742 | 0.1345 | Enriched | 0.0228 |
| TOMM40L | rs4233368 | intron | ENST00000367988.3 | c.276+104C>A |  | 0.2886 | 0.2423 | Enriched | 0.0238 |
| COX11 | rs781766974 | missense | ENST00000299335.3 | c.112C>T | p.Pro38Ser | 0.0094 | 0.0011 | Enriched | 0.0251 |
| MUTYH | rs3219484 | missense | ENST00000531105.1 | c.22G>A | p.Val8Met | 0.0340 | 0.0551 | Depleted | 0.0262 |
| UQCRC2 | rs146974535 | missense | ENST00000268379.4 | c.953A>C | p.Gln318Pro | 0.0020 | 0.0096 | Depleted | 0.0263 |
| ALDOA | . | missense | ENST00000569798.1 | c.908G>A | p.Gly303Asp | 0.0062 | 0.0000 | Enriched | 0.0267 |
| NDUFA1 | . | missense | ENST00000371437.4 | c.67A>T | p.Thr23Ser | 0.0062 | 0.0000 | Enriched | 0.0267 |
| TOMM40L | rs12401322 | upstream gene | ENST00000367988 |  |  | 0.2883 | 0.2456 | Enriched | 0.0271 |
| PRR5L | rs62621409 | missense | ENST00000530639.1 | c.415A>G | p.Thr139Ala | 0.0202 | 0.0367 | Depleted | 0.0275 |
| OGG1 | rs1052133 | missense | ENST00000302003.7 | c.994C>G | p.Pro332Ala | 0.2273 | 0.1851 | Enriched | 0.0288 |
| Gene | Variant rsID^+^ | Type | Transcript ID^#^ | Nucleotide Change^#^ | Amino Acid Change^#^ | MAF Case^%^ | MAF Control^%^ | Direction^&^ | P |
| CPS1 | rs1047891 | missense | ENST00000430249.2 | c.4235C>A | p.Thr1412Asn | 0.3710 | 0.3260 | Enriched | 0.0322 |
| ATXN3 | rs147058331 | missense | ENST00000393287.5 | c.854G>A | p.Arg285Gln | 0.0131 | 0.0044 | Enriched | 0.0326 |
| SHC1 | rs8191979 | missense | ENST00000448116.2 | c.1228A>G | p.Met410Val | 0.0292 | 0.0472 | Depleted | 0.0332 |
| MRPL51 | . | missense | ENST00000229238.3 | c.121C>A | p.Pro41Thr | 0.0000 | 0.0052 | Depleted | 0.0334 |
| PRKAB1 | . | missense | ENST00000541640.1 | c.466A>C | p.Lys156Gln | 0.0000 | 0.0052 | Depleted | 0.0334 |
| CYBB | rs141756032 | missense | ENST00000378588.4 | c.1090G>C | p.Gly364Arg | 0.0000 | 0.0052 | Depleted | 0.0334 |
| ATP5S | rs7152491 | intron | ENST00000311459.7 | c.41-3071T>G |  | 0.5212 | 0.4747 | Enriched | 0.0335 |
| DEPTOR | rs4871827 | missense | ENST00000286234.5 | c.1166G>A | p.Ser389Asn | 0.3145 | 0.3584 | Depleted | 0.0349 |
| SPG7 | rs146397529 | upstream gene | ENST00000268704 |  |  | 0.0338 | 0.0181 | Enriched | 0.0352 |
| PPOX | . | missense | ENST00000367999.4 | c.974A>T | p.His325Leu | 0.0000 | 0.0060 | Depleted | 0.0370 |
| AKAP1 | rs61731968 | missense | ENST00000337714.3 | c.901G>A | p.Glu301Lys | 0.0051 | 0.0000 | Enriched | 0.0385 |
| FIS1 | . | intron | ENST00000435848.1 | c.15-1588A>G |  | 0.0051 | 0.0000 | Enriched | 0.0385 |
| NDUFS2 | rs11576415 | missense | ENST00000392179.4 | c.1054C>G | p.Pro352Ala | 0.0575 | 0.0385 | Enriched | 0.0412 |
| ATP5S | rs2275592 | missense | ENST00000311459.7 | c.53C>T | p.Pro18Leu | 0.5071 | 0.4624 | Enriched | 0.0414 |
| MRPL51 | . | 5 prime UTR | ENST00000229238.3 | c.-31G>T |  | 0.0042 | 0.0000 | Enriched | 0.0418 |
| MFN2 | . | stop gained | ENST00000235329.5 | c.1642G>T | p.Gly548Ter | 0.0042 | 0.0000 | Enriched | 0.0418 |
| ACADM | rs576688757 | upstream gene | ENST00000420607 |  |  | 0.0141 | 0.0052 | Enriched | 0.0419 |
| NARS2 | rs773682065 | missense | ENST00000529880.1 | c.91C>T | p.Arg31Trp | 0.0051 | 0.0000 | Enriched | 0.0431 |
| ACACB | rs546114684 | missense | ENST00000338432.7 | c.4072G>A | p.Gly1358Ser | 0.0051 | 0.0000 | Enriched | 0.0431 |
| COX4I2 | rs11907253 | missense | ENST00000376075.3 | c.482G>A | p.Arg161His | 0.1124 | 0.0835 | Enriched | 0.0435 |
| MPV17 | rs140992482 | missense | ENST00000405076.1 | c.122G>A | p.Arg41Gln | 0.0048 | 0.0000 | Enriched | 0.0441 |
| ACACA | rs151005382 | intron | ENST00000451642.1 | c.-204+5387A>T |  | 0.0095 | 0.0026 | Enriched | 0.0444 |
| COX5B | rs11904110 | upstream gene | ENST00000258424 |  |  | 0.1663 | 0.1346 | Enriched | 0.0445 |
| COQ6 | rs199702211 | 5 prime UTR | ENST00000394026.4 | c.-152T>A |  | 0.0083 | 0.0018 | Enriched | 0.0463 |
| SLC25A4 | . | 3 prime UTR | ENST00000281456.6 | c.*590T>A |  | 0.0040 | 0.0000 | Enriched | 0.0464 |
| CYCS | rs116489458 | 3 prime UTR | ENST00000305786.2 | c.*1758C>T |  | 0.0040 | 0.0000 | Enriched | 0.0464 |
| MTOR | . | frameshift | ENST00000361445.4 | c.7185dupC | p.Tyr2396LeufsTer29 | 0.0040 | 0.0000 | Enriched | 0.0464 |
| APIP | . | intron | ENST00000395787.3 | c.57+574C>A |  | 0.0040 | 0.0000 | Enriched | 0.0464 |
| PARP2 | . | intron | ENST00000250416.5 | c.313-56G>T |  | 0.0040 | 0.0000 | Enriched | 0.0464 |
| PARP2 | . | intron | ENST00000250416.5 | c.364-79G>T |  | 0.0040 | 0.0000 | Enriched | 0.0464 |
| MTAP | . | intron ,NMD transcript | ENST00000404796.2 | c.348-18278T>A |  | 0.0040 | 0.0000 | Enriched | 0.0464 |
| Gene | Variant rsID^+^ | Type | Transcript ID^#^ | Nucleotide Change^#^ | Amino Acid Change^#^ | MAF Case^%^ | MAF Control^%^ | Direction^&^ | P |
| CPT2 | rs375766702 | missense | ENST00000371486.3 | c.1901G>A | p.Arg634Gln | 0.0040 | 0.0000 | Enriched | 0.0464 |
| OMA1 | rs34466938 | missense | ENST00000371226.3 | c.201C>A | p.Asn67Lys | 0.0040 | 0.0000 | Enriched | 0.0464 |
| FAR1 | rs765221802 | missense | ENST00000354817.3 | c.742A>C | p.Asn248His | 0.0040 | 0.0000 | Enriched | 0.0464 |
| MRPL11 | . | missense | ENST00000310999.7 | c.331C>A | p.Leu111Met | 0.0040 | 0.0000 | Enriched | 0.0464 |
| ACAT1 | . | missense | ENST00000265838.4 | c.323T>C | p.Val108Ala | 0.0040 | 0.0000 | Enriched | 0.0464 |
| MRPL51 | . | missense | ENST00000229238.3 | c.208C>A | p.Pro70Thr | 0.0040 | 0.0000 | Enriched | 0.0464 |
| ISCU | rs199905986 | missense | ENST00000311893.9 | c.148G>T | p.Gly50Trp | 0.0040 | 0.0000 | Enriched | 0.0464 |
| ACACB | . | missense | ENST00000377854.5 | c.1684A>G | p.Ser562Gly | 0.0040 | 0.0000 | Enriched | 0.0464 |
| SPTLC2 | . | missense | ENST00000216484.2 | c.1584A>G | p.Ile528Met | 0.0040 | 0.0000 | Enriched | 0.0464 |
| ACSM2A | rs368797865 | missense | ENST00000396104.2 | c.832G>T | p.Ala278Ser | 0.0040 | 0.0000 | Enriched | 0.0464 |
| SOD1 | . | missense | ENST00000270142.6 | c.406A>G | p.Thr136Ala | 0.0040 | 0.0000 | Enriched | 0.0464 |
| SAMM50 | rs35189432 | missense | ENST00000350028.4 | c.680A>C | p.Lys227Thr | 0.0040 | 0.0000 | Enriched | 0.0464 |
| HARS2 | . | missense | ENST00000230771.3 | c.1332G>T | p.Lys444Asn | 0.0040 | 0.0000 | Enriched | 0.0464 |
| PMPCB | . | missense | ENST00000420236.2 | c.952G>T | p.Gly318Cys | 0.0040 | 0.0000 | Enriched | 0.0464 |
| PCCB | rs202247820 | stop gained | ENST00000471595.1 | c.1495C>T | p.Arg499Ter | 0.0040 | 0.0000 | Enriched | 0.0464 |
| ACADM | . | upstream gene | ENST00000420607 |  |  | 0.0040 | 0.0000 | Enriched | 0.0464 |
| TOMM40L | . | upstream gene | ENST00000367988 |  |  | 0.0040 | 0.0000 | Enriched | 0.0464 |
| GFM1 | . | upstream gene | ENST00000486715 |  |  | 0.0040 | 0.0000 | Enriched | 0.0464 |
| APIP | rs3763931 | intron | ENST00000395787.3 | c.57+359C>T |  | 0.1742 | 0.1398 | Enriched | 0.0485 |
| SLC25A37 | rs3736032 | missense | ENST00000519973.1 | c.287G>A | p.Arg96Gln | 0.0847 | 0.1101 | Depleted | 0.0495 |
| APIP | rs3763933 | intron | ENST00000395787.3 | c.57+445C>A |  | 0.1768 | 0.1419 | Enriched | 0.0496 |

^#^Transcript ID, nucleotide, and amino acid changes annotated from Ensembl VEP (Variant Effect Predictor).

^+^Variant rsID from dbSNP144. ^%^Minor allele frequency (MAF) reported is calculated from the corrected observed allele count for each group of the cohort.

Table S7. Nonsynonymous longevity-associated mitonuclear gene variants (p≤0.050).

| **VARIANT IDENTIFIERS** | | | | **MINOR ALLELE FREQUENCY (MAF)** | | | | | **AMINO ACID (AA) SUBSTITUTION DETAILS AND IMPACT PREDICTIONS** | | | | | |
| --- | --- | --- | --- | --- | --- | --- | --- | --- | --- | --- | --- | --- | --- | --- |
| **Gene** | **Variant ID** | **Location_**  **Alleles^A^** | **Type^B^** | **P** | **Enr^C^** | **Case** | **Control** | **Valid^D^** | **AA Change^E^** | **AA Cons^F^** | **Protein Domains** | **AA Evo. Cons^G^** | **Condel^H^** | **CADD^J^** |
| **LRPPRC** | rs146630100 | 2_44116945_ A/T | m | 0.0046 | Enr | 0.0071 | 0.0000 | yes | D1352E | C | RNA-binding | rn, mm, xt, dr | N | U |
| NOX1 | rs142303829 | X_100117243_ G/A | m | 0.0130 | Enr | 0.0786 | 0.0516 | yes | R241C | R | Ferric oxidoreductase, extracellular | None | N | U |
| COX15 | rs2231687 | 10_101473218_ A/G | m | 0.0202 | Enr | 0.2117 | 0.1713 | yes | F374L | R | None | None | N | - |
| ITPR1 | . | 3_4853016_ G/T | m | 0.0215 | Enr | 0.0050 | 0.0000 | yes | R2432L | R | Ion_transport, Cytoplasmic | mm, rn, xt, dr, dm | D | L |
| **LRPPRC** | rs149693840 | 2_44115792_ T/C | m | 0.0215 | Enr | 0.0050 | 0.0000 | yes | S1378G | NC | RNA binding | rn, mm, xt, dr | D | P |
| ALDOA | . | 16_30081259_ G/A | m | 0.0267 | Enr | 0.0062 | 0.0000 | yes | G357D | NC | Beta Sheet | mm, rn, xt, dr, dm, ce, at | D | L |
| OGG1 | rs1052133 | 3_9798773_ C/G | m | 0.0288 | Enr | 0.2273 | 0.1851 | yes | P332A | NC | None | - | N | U |
| CPS1 | rs1047891 | 2_211540507_ C/A | m | 0.0322 | Enr | 0.3710 | 0.3260 | yes | T1412N | NC | Beta-strand, allosteric activation site adjacent | mm, rn,dr, ce | N | U |
| ATXN3 | rs147058331 | 14_92547327_ C/T | m | 0.0326 | Enr | 0.0131 | 0.0044 | yes | R285Q | NC | Coiled-coil | mm, rn, xt, dr, ce | D | L |
| AKAP1 | rs61731968 | 17_55183726_ G/A | m | 0.0385 | Enr | 0.0051 | 0.0000 | yes | E301K | NC | None | None | N | U |
| NDUFS2 | rs11576415 | 1_161182208_ C/G | m | 0.0412 | Enr | 0.0575 | 0.0385 | yes | P352A | NC | NADH-quinone oxidoreductase, metal binding adjacent | mm, rn, xt, dr, dm, ce, at | D | P |
| MFN2 | . | 1_12065914_ G/T | sg | 0.0418 | Enr | 0.0042 | 0.0000 | yes | G548* | R | Cytoplasmic | mm, rn, xt, dr, dm, ce, sp | - | L |
| COX4I2 | rs11907253 | 20_30232673_ G/A | m | 0.0435 | Enr | 0.1124 | 0.0835 | yes | R161H | C | None | None | N | U |
| HARS2 | . | 5_140077534_ G/T | m | 0.0464 | Enr | 0.0040 | 0.0000 | yes | K444N | R | N6-acetyllisine | mm, rn, xt, dr, dm, ce, sp | D | L |
| FAR1 | rs765221802 | 11_13733329_ A/C | m | 0.0464 | Enr | 0.0040 | 0.0000 | yes | N248H | R | NAD-binding,  Cytoplasmic | mm, rn, xt, dr, dm, ce | D | L |
| ISCU | rs199905986 | 12_108958088_ G/T | m | 0.0464 | Enr | 0.0040 | 0.0000 | yes | G50W | R | NifU-like_Nterm – FeS Cluster Assembly | mm, rn, xt, dm, ce, sc | D | L |
| PMPCB | . | 7_102952281_G/T | m | 0.0464 | Enr | 0.0040 | 0.0000 | yes | G423C | NC | None | mm, rn, xt, dr, dm, ce, sc, sp, at | D | L |
| OMA1 | rs34466938 | 1_59004766_ G/T | m | 0.0464 | Enr | 0.0040 | 0.0000 | yes | N67K | R | None | mm, rn | D | U |
| ACACB | . | 12_109617758_ A/G | m | 0.0464 | Enr | 0.0040 | 0.0000 | yes | S562G | NC | ATP-grasp, Biotin Carboxylation | mm, rn, xt, dm, ce, sc, sp | D | P |
| SAMM50 | rs35189432 | 22_44371966_ A/C | m | 0.0464 | Enr | 0.0040 | 0.0000 | yes | K227T | R | Bacterial surface antigen | mm, rn, xt, dr | N | L |
| MTOR | . | 1_11174490_ -/G | fs | 0.0464 | Enr | 0.0040 | 0.0000 | yes | Y2396Lfs*29 | R | PI3K/PI4K, Catalytic Domain, Helix | mm, rn, xt, dr, dm, ce | - | - |
| ACSM2A | rs368797865 | 16_20482949_ G/T | m | 0.0464 | Enr | 0.0040 | 0.0000 | yes | A278S | NC | AMP-binding, helix | None | N | U |
| SPTLC2 | . | 14_77978732_ T/C | m | 0.0464 | Enr | 0.0040 | 0.0000 | yes | I528M | R | Amino-transferase_1_2 | mm, rn, xt, dr, dm, at | N | P |
| ABCB8 | rs139216778 | 7_150730900_ C/T | m | 0.0139 | Dep | 0.0000 | 0.0100 | yes | P139S | NC | None | mm, rn, xt, dr, dm | N | U |
| **VARIANT IDENTIFIERS** | | | | **MINOR ALLELE FREQUENCY (MAF)** | | | | | **AMINO ACID (AA) SUBSTITUTION DETAILS AND IMPACT PREDICTIONS** | | | | | |
| ACADM | . | 1_76200503_ G/A | m | 0.0171 | Dep | 0.0000 | 0.0061 | yes | D172N | NC | Acyl-coA dehydrogenase N -term, helix | mm, rn, dr, ce | N | P |
| PFKM | rs2228500 | 12_48526712_ G/A | m | 0.0195 | Dep | 0.2319 | 0.2762 | yes | R171Q | NC | N-term catalytic PFK | mm, rn | N | U |
| KIF1B | rs2297881 | 1_10397567_ A/G | m | 0.0200 | Dep | 0.0292 | 0.0490 | yes | Y1087C | R | None | mm, rn, xt, dr, dm, ce | D | L |
| UQCRC2 | rs146974535 | 16_21983430_ A/C | m | 0.0263 | Dep | 0.0020 | 0.0096 | yes | Q318P | R | Peptidas_M16_C | rn, at | N | U |
| PRR5L | rs62621409 | 11_36458997_ A/G | m | 0.0275 | Dep | 0.0202 | 0.0367 | yes | T139A | NC | None | mm, rn, st, dr | D | P |
| SHC1 | rs8191979 | 1_154938662_ T/C | m | 0.0332 | Dep | 0.0292 | 0.0472 | yes | M410V | NC | CH1 | mm, rn | N | U |
| CYBB | rs141756032 | X_37663322_ G/C | m | 0.0334 | Dep | 0.0000 | 0.0052 | yes | G364R | R | FAD-binding FR-type, Cytoplasmic | mm, rn, xt | N | L |
| DEPTOR | rs4871827 | 8_121061879_ G/A | m | 0.0349 | Dep | 0.3145 | 0.3584 | yes | S389N | NC | PDZ | mm, rn, xt, dr | N | P |
| SLC25A37 | rs3736032 | 8_23423697_ G/A | m | 0.0495 | Dep | 0.0847 | 0.1101 | yes | R96Q | NC | Solute Carrier Repeat 1 (Solcar) | mm, xt, dr | N | U |
| ATP5S | rs12433794 | 14_50786004_ A/G | m | 0.0071 | Enr | 0.5121 | 0.4537 | - | E18G | R | N-terminal, mito transit peptide | None | N | U |
| PDP1 | . | 8_94935837_ A/T | m | 0.0215 | Enr | 0.0050 | 0.0000 | - | D542V | NC | PPM-type Phosphatase | mm, rn, xt, dr, dm, ce, sc, sp | D | L |
| ALDOA | . | 16_30081399_ G/T | sg | 0.0219 | Enr | 0.0068 | 0.0000 | - | E350* | R | None | - | - | U |
| COX11 | rs781766974 | 17_53045896_ G/A | m | 0.0251 | Enr | 0.0094 | 0.0011 | - | P38S | NC | None | mm,rn | N | U |
| NDUFA1 | . | X_119005941_ A/T | m | 0.0267 | Enr | 0.0062 | 0.0000 | - | T23S | C | Trans-membrane | mm, rn, xt, dr | N | U |
| ATP5S | rs2275592 | 14_50788213_ C/T | m | 0.0414 | Enr | 0.5071 | 0.4624 | - | P18L | NC | N-term Mitochondrial transit peptide | None | N | U |
| NARS2 | rs773682065 | 11_78285443_G/A | m | 0.0431 | Enr | 0.0051 | 0.0000 | - | R31W | NC | None | mm, rn, xt | D | L |
| ACACB | rs546114684 | 12_109670544_ G/A | m | 0.0431 | Enr | 0.0051 | 0.0000 | - | G1358S | NC | None | mm, rn, xt | N | P |
| MPV17 | rs140992482 | 2_27535925_ C/T | m | 0.0441 | Enr | 0.0048 | 0.0000 | - | R41Q | NC | None | mm, rn, dr | D | U |
| MRPL51 | . | 12_6601616_ G/T | m | 0.0464 | Enr | 0.0040 | 0.0000 | - | P70T | NC | Helix | mm, rn, xt, dr, dm, ce | D | L |
| SOD1 | . | 21_33040832_ A/G | m | 0.0464 | Enr | 0.0040 | 0.0000 | - | T136A | NC | Disulfide bond, Helix | mm, rn | N | U |
| CPT2 | rs375766702 | 1_53679191_ G/A | m | 0.0464 | Enr | 0.0040 | 0.0000 | - | R634Q | NC | Mitochondrial matrix | mm, rn, xt | N | U |
| MRPL11 | . | 11_66204717_G/T | m | 0.0464 | Enr | 0.0040 | 0.0000 | - | L111M | R | None | mm, rn | N | U |
| PCCB | rs202247820 | 3_136047696_ C/T | sg | 0.0464 | Enr | 0.0040 | 0.0000 | - | R499* | R | C-term carboxy-terminase | mm, rn, xt, dr, dm, ce | P | L |
| ACAT1 | . | 11_108005032_T/C | m | 0.0464 | Enr | 0.0040 | 0.0000 | - | V108A | C | Helix | None | N | U |
| OXA1L | rs8572 | 14_23236524_ C/T | m | 0.0029 | Dep | 0.1465 | 0.2013 | - | V104A | C | Mitochondrial Intermembrane | mm, rn, ce | N | U |
| NDUFS1 | . | 2_207009640_ T/C | m | 0.0088 | Dep | 0.0000 | 0.0070 | - | E283G | R | 4Fe-4S Mo/W bis-MGD-type | mm, rn, xt, dr, dm, ce, at | D | L |
| TOMM20 | . | 1_235275399_ C/T | m | 0.0088 | Dep | 0.0000 | 0.0070 | - | A140T | NC | Cytoplasmic | mm, rn, xt | N | U |
| SUCLA2 | rs771276854 | 13_48528644_ C/T | m | 0.0137 | Dep | 0.0010 | 0.0087 | - | R284H | C | ATP-grasp, Succinate-CoA Ligase ADP-forming subunit | mm, rn, xt, dr, dm, ce, sc, sp, at | N | P |
| AACS | rs374884365 | 12_125587273_G/A | m | 0.0171 | Dep | 0.0000 | 0.0061 | - | V207M | NC | AMP-binding extracellular | mm, rn, xt, dr, ce | D | L |
| UQCC | . | 20_33934999_ C/T | m | 0.0171 | Dep | 0.0000 | 0.0061 | - | E181K | NC | None | mm, rn, xt, dr, sp | D | L |
| **VARIANT IDENTIFIERS** | | | | **MINOR ALLELE FREQUENCY (MAF)** | | | | | **AMINO ACID (AA) SUBSTITUTION DETAILS AND IMPACT PREDICTIONS** | | | | | |
| GLS2 | rs2657879 | 12_56865338_ A/G | m | 0.0200 | Dep | 0.1371 | 0.1740 | - | L581P | NC | ANK2 repeats | mm, rn | N | U |
| MUTYH | rs3219484 | 1_45800156_ C/T | m | 0.0262 | Dep | 0.0340 | 0.0551 | - | V22M | NC | None | mm, rn, dr | N | U |
| PRKAB1 | . | 12_120112193_A/C | m | 0.0334 | Dep | 0.0000 | 0.0052 | - | K156Q | NC | Glycogen binding, 5'AMP-activated kinase subunit | mm, rn, dm | N | P |
| MRPL51 | . | 12_6602097_ G/T | m | 0.0334 | Dep | 0.0000 | 0.0052 | - | P41T | NC | None | mm, rn, xt | N | P |
| PPOX | . | 1_161139801_ A/T | m | 0.0370 | Dep | 0.0000 | 0.0060 | - | H325L | NC | Amino-oxidase | None | N | U |

Table is ordered by p-value, enrichment, and validation. ^A^Location_Alleles: Chromosome_Position_ REF/ALT; ^B^Type: m=missense, fs = frameshift, sg = stop gain; ^C^Direction of enrichment: (Enr) or depletion (Dep) is with respect to cases (centenarians) in the cohort, MAF reported is corrected for observed alleles post sequencing; ^D^Validated by genotyping: “-” = not validated (lower portion of the table shaded in gray); ^E^AA Change: Residue number in between Reference AA and Alternative AA single letter symbol; ^F^AA [Biochemical] Conservation: C= Conserved, NC = Not Conserved, R=Radical; ^G^AA Evolutionary Conservation: rn = *Rattus norvegicus* (Rat), mm = *Mus musculus* (mouse), xt = *Xenopus tropicalis* (frog), dr = *Danio rerio* (fish), dm = *Drosophila melanogaster* (fly), ce=*Caenorhabditis elegans* (worm), at = *Arabidopsis thaliana* (plant); ^H^Condel prediction: D = Deleterious, N = Neutral; ^J^CADD prediction: L = Probably damaging (Likely), P = Possibly damaging, U = Unlikely damaging

Table S8. Validated longevity-associated nonsynonymous variants with high damage potential.

| **Variant Identifiers** | | | | **Minor Allele Frequency** | | | | | | | | | **Amino Acid Substitution and Predictions** | | | | | | |
| --- | --- | --- | --- | --- | --- | --- | --- | --- | --- | --- | --- | --- | --- | --- | --- | --- | --- | --- | --- |
| **Gene** | **Variant ID** | **Location_**  **REF/ALT^A^** | **Type^B^** | **P** | **Enr^C^** | **Case** | **Control** | **1000G** | **Gnom AD** | **ExAc** | **DiscovEHR** | **GC^D^** | **AA Change^E^** | **Protein Domains** | **AA Con^F^** | **Vert Con^G^** | **Inv Con^H^** | **C^J^** | **CADD^K^** |
| ITPR1 | . | 3_4853016_G/T | m | 0.021 | Enr | 0.005 | 0.000 | - | - | - | - | H | R2432L | Cytoplasmic, Ion_transport | R | H | yes | D | L |
| **LRPPRC** | rs149693840 | 2_44115792_T/C | m | 0.021 | Enr | 0.005 | 0.000 | - | 0.000 | 0.000 | <0.001 | H | S1378G | RNA binding | NC | H | no | D | P |
| ALDOA | . | 16_30081259_G/A | m | 0.027 | Enr | 0.006 | 0.000 | - | - | - | - | H | G357D | Beta Sheet | R | H | yes* | D | L |
| MFN2 | . | 1_12065914_G/T | sg | 0.042 | Enr | 0.004 | 0.000 | - | - | - | <0.001 | L | G548* | Cytoplasmic | R | H | yes** | - | L |
| SPTLC2 | . | 14_77978732_T/C | m | 0.046 | Enr | 0.004 | 0.000 | - | - | - | - | L | I528M | Aminotransferase_1_2 domain | R | W | yes | N | P |
| ACACB | . | 12_109617758_A/G | m | 0.046 | Enr | 0.004 | 0.000 | - | - | - | - | M | S562G | ATP-grasp, Biotin Carboxylation, beta chain | NC | H | yes** | D | P |
| SAMM50 | rs35189432 | 22_44371966_A/C | m | 0.046 | Enr | 0.004 | 0.000 | - | 0.000 | 0.000 | <0.001 | H | K227T | Bacterial surface antigen | R | H | no | N | L |
| FAR1 | rs765221802 | 11_13733329_A/C | m | 0.046 | Enr | 0.004 | 0.000 | - | 0.000 | 0.000 | - | H | N248H | Cytoplasmic, NAD-binding_4 | R | H | yes* | D | L |
| HARS2 | . | 5_140077534_G/T | m | 0.046 | Enr | 0.004 | 0.000 | - | - | - | - | M | K444N | N6-acetyllisine | R | W | yes** | D | L |
| ISCU | rs199905986 | 12_108958088_G/T | m | 0.046 | Enr | 0.004 | 0.000 | 0.000 | 0.000 | - | - | H | G50W | NifU_N | R | H | yes** | D | L |
| PMPCB | . | 7_102952281_G/T | m | 0.046 | Enr | 0.004 | 0.000 | - | - | - | - | L | G423C | None | NC | H | yes** | D | L |
| ATXN3 | rs147058331 | 14_92547327_C/T | m | 0.033 | Enr | 0.013 | 0.004 | - | 0.000 | 0.000 | <0.001 | M | R285Q | Coiled-coil | NC | H | yes | D | L |
| NDUFS2 | rs11576415 | 1_161182208_C/G | m | 0.041 | Enr | 0.057 | 0.038 | 0.049 | 0.075 | 0.078 | 0.093 | M | P352A | CI_49kDa | NC | H | yes* | D | P |
| ACADM | . | 1_76200503_G/A | m | 0.017 | Dep | 0.000 | 0.006 | - | - | - | - | M | D172N | Acyl-coA dehydrogenase N -term, helix | NC | H | yes | N | P |
| KIF1B | rs2297881 | 1_10397567_A/G | m | 0.020 | Dep | 0.029 | 0.049 | 0.040 | 0.033 | 0.033 | 0.023 | M | Y1087C | None | R | H | yes* | D | L |
| PRR5L | rs62621409 | 11_36458997_A/G | m | 0.027 | Dep | 0.020 | 0.037 | 0.019 | 0.039 | 0.039 | 0.040 | M | T139A | None | NC | H | no | D | P |
| CYBB | rs141756032 | X_37663322_G/C | m | 0.033 | Dep | 0.000 | 0.005 | 0.002 | 0.004 | 0.004 | 0.005 | H | G364R | FAD-binding FR-type, Cytoplasmic | R | H | no | N | L |
| DEPTOR | rs4871827 | 8_121061879_G/A | m | 0.035 | Dep | 0.315 | 0.358 | 0.300 | 0.331 | - | 0.331 | M | S389N | PDZ | R | H | no | N | P |

Table is ordered by p-value, enrichment, and validation. ^A^Location_REF/ALT: Chromosome_Position_Reference allele/Alternative allele (human reference genome: hg19/GRCh37); ^B^Type of polymorphism: missense (m), frameshift (fs), stop gain (sg), synonymous (s), upstream (up), downstream (down), 5UTR (5 prime untranslated region), 3UTR (3 prime untranslated region), intronic (i), non-mediated disjunction (NMD); ^C^Enriched (Enr) or Depleted (Dep) in centenarians; ^D^Genotyping Confidence (GC): High (H), Moderate (M), Low (L); ^E^Amino acid (AA) change: Residue number in between Reference AA and Alternative AA single letter symbol; ^F^AA Biochemical Conservation (AA Con): R = Radical, NC = Non-conserved, C = Conserved; ^G^Vertebrate Conservation (Vert Con): H = High, W = Weak, Peak level from UCSC genome browser 100 vertebrate multiz alignment conservation track; ^H^Invertebrate Conservation (Inv Con): Residue conservation in invertebrate model organisms (*Drosophila melanogaster*, *Caenorhabditis elegans*, and *Sacchromyces cerevisiae* or *pombes*), yes** = conservation in all three, yes* = conservation in 2, yes = conservation in 1, no = not conserved in listed invertebrates; ^J^Condel ('C) prediction: D = Deleterious, N = Neutral; ^K^CADD prediction: L = Probably damaging (Likely), P = Possibly damaging, U = Unlike
